# Supplementary material for: Prodrug florfenicol amine is activated by intrinsic resistance to target Mycobacterium abscessus
Source: Nat Microbiol. 2025 Oct 30;10(11):2875–91. doi: 10.1038/s41564-025-02147-9 (PMC12578646; doi:10.1038/s41564-025-02147-9)
Supplement: Supplementary file 1 — Supplementary Tables 1–11, chemistry materials and methods, source data inventory, compound characterization, source data for blots and uncropped images (Figs. 1–4 and original gel/plate images) and references. [file 41564_2025_2147_MOESM1_ESM.pdf]

nature microbiology

Supplementary information

https://doi.org/10.1038/s41564-025-02147-9

Prodrug florfenicol amine is activated by intrinsic resistance to target *Mycobacterium abscessus*

In the format provided by the authors and unedited

Supporting information for:

Prodrug florfenicol amine is activated by intrinsic resistance to target *Mycobacterium abscessus*

Gregory A. Phelps[1,2,†], Sinem Kurt[3,†], Alexander R. Jenner[1,4], Shelby M. Anderson[1], Thalina D. Jayasinghe[1], Elizabeth C. Griffith[1], Carl W. Thompson[1], Lei Yang[1], Basil Wicki[5], Frederick K. Bright[5], Victoria Loudon[1], William C. Wright[6], Ashish Srivastava[4], Amarinder Singh[4], Bhargavi Thalluri[4], Hyunseo Park[4], Robin B. Lee[1], Anna K. Wright[1], Oliver Grant-Chapman[2], Daryl K. Conner[7], Brennen T. Troyer[7], Amy Iverson[8], Jason Ochoado[1], Vishwajeeth R. Pagala[9], Long Wu[9], Stephanie Byrum[9], Yingxue Fu[9], Zu-Fei Yuan[9], Anthony A. High[9], Bettina Schulthess[3,10], Jason W. Rosch[8], Paul Geeleher[6], Sven N. Hobbie[11], Lucas Boeck[5,12], Bernd Meibohm[4], Andres Obregon-Henao[7], Peter Sander[3,10,*], Richard E. Lee[1,*]

[1]Department of Chemical Biology and Therapeutics, St. Jude Children's Research Hospital, Memphis, TN, 38105, USA

[2]Graduate School of Biomedical Sciences, St. Jude Children's Research Hospital, Memphis TN, 38103, USA

[3]Institute of Medical Microbiology, University of Zurich, Gloriastrasse 28/30, CH-8006, Zurich, Switzerland

[4]Department of Pharmaceutical Sciences, University of Tennessee Health Science Center, Memphis, TN, 38163, USA

[5]Department of Biomedicine, University of Basel, Basel, Switzerland

[6]Department of Computational Biology, St. Jude Children's Research Hospital, Memphis, TN, 38105, USA

[7]NTM Center, Mycobacteria Research Laboratory, Department of Microbiology, Immunology, and Pathology, Colorado State University, Fort Collins, CO, 80523, USA

[8]Department of Host-Microbe Interactions, St. Jude Children's Research Hospital, Memphis, TN, 38105, USA

[9]Center for Proteomics and Metabolomics, St. Jude Children's Research Hospital, Memphis, TN, 38105, USA

[10]National Reference Center for Mycobacteria, Gloriastrasse 28/30, CH-8006 Zurich, Switzerland

[11]Division of Clinical Bacteriology and Mycology, University Hospital Basel, Basel, Switzerland

[12]Pulmonary Medicine, University Hospital Basel, Switzerland

[†]These authors contributed equally

*Correspondence should be addressed to P.S. (psander@imm.uzh.ch) and R.E.L. (Richard.Lee@StJude.org).

Contents

Supplemental Tables 3

Table S1| Chloramphenicol activity is reduced by Cat (MAB_2989) in *Mycobacterium abscessus*. 3

Table S2| Mutations associated with resistance to florfenicol amine. 4

Table S3| Kinetic parameters of Eis2 for known substrates and amine phenicols. 6

Table S4| *M. smegmatis* cell-free translation inhibition parameters. 7

Table S5| Rapidly growing non-tuberculous mycobacteria (NTM) clinical isolate susceptibility data. 8

Table S6| Antimicrobial activity of florfenicol amine-resistant mutants after 14 days. 10

Table S7| Florfenicol amine lacks mitochondrial protein synthesis inhibition activity and cytotoxicity. 11

Table S8| Metabolic stability of florfenicol amine. 12

Table S9| Summary of metabolites of florfenicol amine detected in hepatocyte stability assay samples. 13

Table S10| Pharmacokinetic parameters for florfenicol amine in BALB/c mice after different routes of administration. 14

Table S11| List of genetically engineered *M. abscessus* and *M. smegmatis* strains and primers used. 15

Chemistry Materials and Methods 16

Source Data Inventory 19

Compound characterization 20

Source Data for Blots and Uncropped Images 24

Figure S1| Southern Blot analysis confirms the deletion of cat (MAB_2989) from the genome of *M. abscessus*. 24

Figure S2| Southern blot analysis of complementation of *cat* in *M. abscessus*. 25

Figure S3| Gel Image of colony PCR of recombinant *M. smegmatis* pOLYG-*aac(3)IV-cat*. 26

Figure S4| Gel Image of colony PCR of recombinant *M. smegmatis* pOLYG-*aac(3)IV-eis2*. 27

Original, uncropped gel image corresponding to Figure S1. 28

Original, uncropped gel image corresponding to Figure S2. 29

Original, uncropped gel image corresponding to Figure S3. 30

Original, uncropped gel image corresponding to Figure S4. 31

Original agar plate images corresponding to Extended Data Figure 2, panel a. 32

Original agar plate images corresponding to Extended Data Figure 2, panel b. 33

Original agar plate images corresponding to Extended Data Figure 2, panel c. 34

Supplementary References 35

Supplemental Tables

Table S1| Chloramphenicol activity is reduced by Cat (MAB_2989) in *Mycobacterium abscessus*. Minimal Inhibitory Concentration (MIC) of *M. smegmatis* and *M. abscessus* clinical isolates against chloramphenicol (CAM) and amikacin (AMK).

	**Minimum inhibitory concentration ($\mu g\ mL^{-1}$)**	
**Strain**	**CAM**	**AMK**
M. smegmatis mc2- 155	16	0.125
M. smegmatis pOLYG-aac(3)IV	16	0.125
M. smegmatis pOLYG-aac(3)IV-*cat*	128	0.125
M. abscessus subsp. *abscessus* V0077	2048	4
M. abscessus subsp. *massiliense* V0074	1024	4
M. abscessus subsp. *bolletti* V0082	2048	8
M. abscessus subsp. *massiliense* V0084*	256	4
M. abscessus subsp. *massiliense* V0199*	128	4
M. abscessus subsp. *massiliense* V0204*	256	4
M. abscessus subsp. *bolletti* V0233*	256	0.5-1

*contains frameshift mutation in *cat*

Table S2| Mutations associated with resistance to florfenicol amine. Table displaying the sample name, sequencing platform, concentration (µg mL^{-1}) of florfenicol amine ($FF-NH_2$)used for mutant selection, type of mutation observed, gene where mutation occurred, specific nucleotide change, specific amino acid change, and sequence read archive (SRA) accession details.

Sample Name	Sequencing Platform	$FF-NH_2$ Selection Concentration (µg mL^{-1})	Type	Gene	Nucleotide Change	Amino Acid Change	SRA Accession
WGS_FFA256_Mut1	WGS	256	Deletion	*eis2*	358delG	Glu120fs	SAMN42914275
WGS_FFA256_Mut2	WGS	256	SNP	*eis2*	765C>A	Tyr255*	SAMN42914276
WGS_FFA256_Mut3	WGS	256	SNP	*eis2*	852C>A	Tyr284*	SAMN42914277
WGS_FFA256_Mut4	WGS	256	Deletion	*eis2*	164delA	Lys55fs	SAMN42914278
WGS_FFA256_Mut5	WGS	256	Deletion	*eis2*	164delA	Lys55fs	SAMN42914279
WGS_FFA256_Mut6	WGS	256	Replacement	*eis2*	266delinsGC	His89fs	SAMN42914280
WGS_FFA256_Mut7	WGS	256	Insertion	*eis2*	279dupG	Leu94fs	SAMN42914281
WGS_FFA256_Mut8	WGS	256	Deletion	*eis2*	164delA	Lys55fs	SAMN42914282
WGS_FFA256_Mut9	WGS	256	Insertion	*eis2*	279dupG	Leu94fs	SAMN42914283
WGS_FFA256_Mut10	WGS	256	Deletion	*eis2*	164delA	Lys55fs	SAMN42914284
WGS_FFA128_Mut1	WGS	128	SNP	*whiB7*	110T>C	Leu37Pro	SAMN42914285
WGS_FFA128_Mut2	WGS	128	Insertion	*eis2*	279dupG	Leu94fs	SAMN42914286
WGS_FFA128_Mut3	WGS	128	SNP	*whiB7*	151G>A	Ala51Thr	SAMN42914287
WGS_FFA128_Mut4	WGS	128	SNP	*eis2*	679T>G	Tyr227Asp	SAMN42914288
WGS_FFA128_Mut5	WGS	128	SNP	*eis2*	908G>T	Arg303Leu	SAMN42914289
WGS_FFA128_Mut6	WGS	128	Insertion	*eis2*	279dupG	Leu94fs	SAMN42914290
WGS_FFA64_Mut1	WGS	64	Insertion	*whiB7*	207dupG	Thr70fs	SAMN42914291
WGS_FFA64_Mut2	WGS	64	Deletion	*eis2*	164delA	Lys55fs	SAMN42914292
WGS_FFA64_Mut3	WGS	64	Deletion	*whiB7*	70_73delTTCG	Phe24fs	SAMN42914293
WGS_FFA64_Mut4	WGS	64	SNP	*eis2*	773C>G	Thr258Arg	SAMN42914294
WGS_FFA64_Mut5	WGS	64	Deletion	*whiB7*	227del_MAB_3504	Lys76del	SAMN42914295
WGS_FFA64_Mut6	WGS	64	SNP	*eis2*	679T>G	Tyr227Asp	SAMN42914296
Sanger_Eis2-1	Sanger	64	Deletion	*eis2*	567delC	Glu190fs	
Sanger_Eis2-2	Sanger	64	Deletion	*eis2*	508delG	Ala170fs	
Sanger_Eis2-3	Sanger	64	Deletion	*eis2*	126delC	Glu43fs	
Sanger_Eis2-4	Sanger	64	SNP	*eis2*	358G>A	Glu120Lys	
Sanger_Eis2-8	Sanger	64	Deletion	*eis2*	274delC	Arg92fs	
Sanger_Eis2-9	Sanger	64	SNP	*eis2*	3G>T	No Start	
Sanger_Eis2-10	Sanger	64	SNP	*eis2*	559C>T	Arg187Cys	
Sanger_Eis2-11	Sanger	64	Deletion	*eis2*	164delA	Lys55fs	
Sanger_Eis2-12	Sanger	64	SNP	*eis2*	268C>T	Arg90Trp	
Sanger_Eis2-15	Sanger	64	Deletion	*eis2*	279delG	Leu94fs	
Sanger_Eis2-16	Sanger	64	SNP	*eis2*	398C>G	Thr133Arg	
Sanger_Eis2-17	Sanger	64	Insertion	*eis2*	217_218insA	Ala73fs	
Sanger_Eis2-18	Sanger	64	SNP	*eis2*	308T>G	Leu103Trp	
Sanger_Eis2-20	Sanger	64	Insertion	*eis2*	279dupG	Leu94fs	
Sanger_Eis2-22	Sanger	64	SNP	*eis2*	1236G>T	*412Tyr	
Sanger_Eis2-49	Sanger	64	SNP	*eis2*	248T>A	Val83Asp	
Sanger_Eis2-52	Sanger	64	Deletion	*eis2*	164delA	Lys55fs	
Sanger_Eis2-53	Sanger	64	Insertion	*eis2*	279dupG	Leu94fs	
Sanger_Eis2-55	Sanger	64	SNP	*eis2*	46G>T	Glu16*	
Sanger_Eis2-56	Sanger	64	SNP	*eis2*	547G>A	Gly183Arg	
Sanger_Eis2-57	Sanger	64	Deletion	*eis2*	260delC	Pro87fs	
Sanger_Eis2-58	Sanger	64	Deletion	*eis2*	164delA	Lys55fs	
Sanger_Eis2-59	Sanger	64	SNP	*eis2*	257C>A	Ser86*	
Sanger_Eis2-60	Sanger	64	SNP	*eis2*	248T>A	Val83Asp	
Sanger_Eis2-61	Sanger	64	Deletion	*eis2*	757delC	Arg253fs	
Sanger_Eis2-62	Sanger	64	Deletion	*eis2*	164delA	Lys55fs	
Sanger_Eis2-64	Sanger	64	SNP	*eis2*	248T>A	Val83Asp	
Sanger_Eis2-65	Sanger	64	SNP	*eis2*	383G>T	Gly128Val	
Sanger_Eis2-66	Sanger	64	Insertion	*eis2*	824dupG	Ala276fs	
Sanger_Eis2-67	Sanger	64	SNP	*eis2*	773C>A	Thr258Lys	
Sanger_Eis2-68	Sanger	64	Deletion	*eis2*	447delC	Thr150fs	
Sanger_Eis2-69	Sanger	64	Insertion	*eis2*	279dupG	Leu94fs	
Sanger_Eis2-70	Sanger	64	Deletion	*eis2*	270delG	Arg91fs	
Sanger_Eis2-71	Sanger	64	Insertion	*eis2*	279dupG	Leu94fs	
Sanger_Eis2-73	Sanger	64	Insertion	*eis2*	1225dupG	Asp409fs	
Sanger_Eis2-74	Sanger	64	SNP	*eis2*	418G>T	Gly140*	
Sanger_Eis2-75	Sanger	64	Deletion	*eis2*	351delC	Phe117fs	
Sanger_Eis2-76	Sanger	64	Deletion	*eis2*	164delA	Lys55fs	

Sanger_Eis2-77	Sanger	64	SNP	*eis2*	635T>C	Val212Ala	
Sanger_Eis2-78	Sanger	64	SNP	*eis2*	635T>C	Val212Ala	
Sanger_Eis2-79	Sanger	64	SNP	*eis2*	646G>T	Glu216*	
Sanger_Eis2-80	Sanger	64	Insertion	*eis2*	567dupC	Glu190fs	
Sanger_Eis2-81	Sanger	64	Deletion	*eis2*	536delC	Pro179fs	
Sanger_Eis2-82	Sanger	64	SNP	*eis2*	136C>T	Arg46Trp	
Sanger_Eis2-84	Sanger	64	SNP	*eis2*	136C>T	Arg46Trp	
Sanger_Eis2-85	Sanger	64	SNP	*eis2*	835C>T	Gln279*	
Sanger_Eis2-86	Sanger	64	SNP	*eis2*	559C>T	Arg187Cys	
Sanger_Eis2-87	Sanger	64	SNP	*eis2*	847C>T	Arg283Cys	
Sanger_Eis2-88	Sanger	64	Deletion	*eis2*	164delA	Lys55fs	
Sanger_Eis2-89	Sanger	64	Deletion	*eis2*	164delA	Lys55fs	
Sanger_Eis2-90	Sanger	64	SNP	*eis2*	680A>G	Tyr227Cys	
Sanger_Eis2-91	Sanger	64	SNP	*eis2*	848G>T	Arg283Leu	
Sanger_Eis2-92	Sanger	64	Deletion	*eis2*	164delA	Lys55fs	
Sanger_Eis2-93	Sanger	64	Deletion	*eis2*	164delA	Lys55fs	
Sanger_Eis2-94	Sanger	64	Deletion	*eis2*	164delA	Lys55fs	
Sanger_Eis2-95	Sanger	64	Deletion	*eis2*	973_1010del	Glu325fs	
Sanger_Eis2-96	Sanger	64	SNP	*eis2*	136C>T	Arg46Trp	
Sanger_WhiB7-25	Sanger	64	Deletion	*whiB7*	34delG	Ala12fs	
Sanger_WhiB7-26	Sanger	64	Deletion	*whiB7*	185delG	Gly62fs	
Sanger_WhiB7-27	Sanger	64	Deletion	*whiB7*	34delG	Ala12fs	
Sanger_WhiB7-28	Sanger	64	SNP	*whiB7*	175G>A	Gly59Arg	
Sanger_WhiB7-29	Sanger	64	SNP	*whiB7*	47A>C	His16Pro	
Sanger_WhiB7-30	Sanger	64	Deletion	*whiB7*	34delG	Ala12fs	
Sanger_WhiB7-32	Sanger	64	SNP	*whiB7*	184G>T	Gly62Cys	
Sanger_WhiB7-33	Sanger	64	SNP	*whiB7*	184G>T	Gly62Cys	
Sanger_WhiB7-34	Sanger	64	SNP	*whiB7*	190G>T	Glu64*	
Sanger_WhiB7-38	Sanger	64	SNP	*whiB7*	185G>C	Gly62Ala	
Sanger_WhiB7-41	Sanger	64	SNP	*whiB7*	184G>T	Gly62Cys	
Sanger_WhiB7-45	Sanger	64	Insertion	*whiB7*	228dupG	Pro77fs	
Sanger_WhiB7-48	Sanger	64	SNP	*whiB7*	184G>T	Gly62Cys	

SNP, single nucleotide polymorphism; WGS, whole-genome sequencing.

Table S3| Kinetic parameters of Eis2 for known substrates and amine phenicols. Table containing the compound name, Michaelis constant (K_M), V_{max}, catalytic constant (k_{cat}), and specificity constant ($k_{cat}\ K_M^{-1}$). Compounds tested: florfenicol (FF), florfenicol amine ($FF\text{-}NH_2$), chloramphenicol (CAM), chloramphenicol amine ($CAM\text{-}NH_2$), thiamphenicol (TAM), thiamphenicol amine ($TAM\text{-}NH_2$), amikacin (AMK), and hygromycin B. Parameters and error (s.d.) are calculated from *n* = 3 biological replicates.

**Compound**	**K_M (µM)**	**V_{max} (µM s^{-1})**	**k_{cat} (s^{-1})**	**$k_{cat}\ K_M^{-1}$ ($M^{-1}\ s^{-1}$)**
FF	Not Active	Not Active	Not Active	Not Active
$FF\text{-}NH_2$	1755 ± 230	0.3 ± 0.0	11.7 ± 0.9	$6.7 \times 10^3 \pm 5.1 \times 10^2$
CAM	Not Active	Not Active	Not Active	Not Active
$CAM\text{-}NH_2$	1002 ± 90	0.2 ± 0.0	9.1 ± 0.3	$9.1 \times 10^3 \pm 5.0 \times 10^2$
TAM	Not Active	Not Active	Not Active	Not Active
$TAM\text{-}NH_2$	371.5 ± 9	0.2 ± 0.0	6.5 ± 0.2	$1.8 \times 10^4 \pm 2.7 \times 10^2$
AMK	232.6 ± 26	0.1 ± 0.0	4.7 ± 0.5	$2.0 \times 10^4 \pm 3.2 \times 10^2$
Hygromycin B	210.0 ± 22	0.2 ± 0.0	6.3 ± 0.4	$3.1 \times 10^4 \pm 4.5 \times 10^3$

Table S4| *M. smegmatis* cell-free translation inhibition parameters. *In vitro* translation inhibition activity of florfenicol (FF), florfenicol acetyl (FF-ac), florfenicol amine (FF-NH_2), chloramphenicol (CAM), and amikacin (AMK), expressed as absolute (Abs) IC_{50} and the corresponding goodness of fit values (R^2) for the dose-response curves, using *M. smegmatis* SZ380 S30 extract. Parameters calculated from *n* = 3 biological replicates.

**Compound**	**Abs. IC_{50} (µM)**	**R^2**
FF	16.9	0.99
FF-ac	39.2	0.99
FF-NH_2	> 800	NA
CAM	20.2	0.99
AMK	0.2	0.66

Table S5| Rapidly growing non-tuberculous mycobacteria (NTM) clinical isolate susceptibility data. Minimal inhibitory concentration (MIC) values for amikacin (AMK), clarithromycin (CLR), florfenicol (FF), and florfenicol amine (FF-NH_2) against a panel of rapidly growing NTM clinical isolates. Relevant mutations associated with drug-resistance are described. Closest orthologue and percentage were collected by NCBI protein BLAST search of the *M. abscessus* Eis2 amino acid sequence against each bacterial organism. MIC determinations were performed by broth microdilution according to CLSI guidelines and reported as the range of *n* = 2 biological experiments. FF/FF-NH_2 ratio was determined by MIC of FF divided by MIC of FF-NH_2. ND, not determined.

			MIC (μg mL^{-1})				
**Strain**	**Allele**	**Closest Eis2* orthologue (%)**	**AMK**	**CLR**	**FF**	**FF-NH_2**	**FF/FF-NH_2 MIC Ratio**
M. abscessus subsp. *abscessus* V0077	*erm*(41) T28C	99.5	2-4	0.25	256	32	8
M. abscessus subsp. *abscessus* V0078	*erm*(41) T28	99.5	4-8	>16	512	32	16
M. abscessus subsp. *abscessus* V0131	*erm*(41) T28	99.5	2	>16	128-256	32	4-8
M. abscessus subsp. *abscessus* V0140	*rrs* A1408G / erm(41) T28	99.5	>32	16	256	16-32	8-16
M. abscessus subsp. *abscessus* V0141	*rrs* A1408G / erm(41) T28C	99.5	>32	0.25	256	32	8
M. abscessus subsp. *abscessus* V0173	*rrs* A1408G/ *cat* R135L / *erm*(41) T28	99.5	>32	16	64-128	16-32	4-8
M. abscessus subsp. *massiliense* V0005	*rrl* A2058C / Δ*erm*(41)	97.76	16	>16	128	32	4
M. abscessus subsp. *massiliense* V0158	Δ*erm*(41)	97.76	2-4	0.25	512	64	8
M. abscessus subsp. *massiliense* V0199	*cat* fs / Δ*erm*(41)	97.76	4	0.5-1	512	64	8
M. abscessus subsp. *massiliense* V0204	*cat* fs / Δ*erm*(41)	97.76	4	0.25-0.5	512	64	8
M. abscessus subsp. *massiliense* V0074	Δ*erm*(41)	97.76	4	0.25	512	32-64	8-16
M. abscessus subsp. *massiliense* V0084	*cat* fs / Δ*erm*(41)	97.76	2-4	0.25	512	32-64	8-16
M. abscessus subsp. *bolletii* V0218	*erm*(41) T28	84.43	2	>16	512	64	8
M. abscessus subsp. *bolletii* V0219	*erm*(41) T28	84.43	1-2	>16	256	32	8
M. abscessus subsp. *bolletii* V0232	*erm*(41) T28	84.43	2	>16	256-512	32-64	8-16
M. abscessus subsp. *bolletii* V0233	*cat* 8 non-syn mutations / *erm*(41) T28	84.43	0.5-1	>16	512	64	8
M. abscessus subsp. *bolletii* V0080	*erm*(41) T28	84.43	2	>16	512	64	8
M. abscessus subsp. *bolletii* V0082	*erm*(41) T28	84.43	8	>16	512	64	8
M. chelonae V0249	Δ*erm*(41)	84.7	16-32	0.5-1	512	8-16	32-64
M. chelonae V0250	Δ*erm*(41)	84.7	16	1-2	256-512	16-32	16-32
M. chelonae V0251	Δ*erm*(41)	84.7	32	0.5-1	512	32	16
M. chelonae V0252	Δ*erm*(41)	84.7	16-32	0.5-1	512	32	16
M. chelonae V0253	Δ*erm*(41)	84.7	16	0.5-2	256	32	8
M. chelonae V0254	Δ*erm*(41)	84.7	16-32	1-4	256-512	64	4-8
M. saopaulense V0256		82.97	4-8	0.625-0.125	128	32	4

M. mucogenicum V0261		64.41	1	ND	>256	>256	ND
M. cosmeticum V0257		64.81	0.5-4	ND	16-32	256	0.0625-0.125
M. cosmeticum V0258		64.81	0.25-1	ND	16-32	256	0.0625-0.125
M. peregrinum V0267		34.8	0.125-0.25	ND	64	128-256	0.25-0.5
M. peregrinum V0268		34.8	0.125	ND	32	128	0.25
M. farcinogenes V0270		34.62	0.5	ND	64-128	512	0.125-0.25
M. farcinogenes V0271		34.62	0.5	ND	64-128	512	0.125-0.25
M. fortuitum V0264		31.02	0.5	ND	64-128	>256	<0.5
M. fortuitum V0265		31.02	1	ND	128-256	256	0.5-1

Table S6| Antimicrobial activity of florfenicol amine-resistant mutants after 14 days. Minimal inhibitory concentration (MIC) values for select antibiotics against a *M. abscessus* WT, *M. abscessus* Δ*whiB7*, *M. abscessus* Δ*eis2*, and two spontaneously generated florfenicol amine (FF-NH_2)-resistant *M. abscessus* mutants. MIC determinations were performed by broth microdilution according to CLSI guidelines and read at 14-days. Fold-change (FC) ratios were determined by dividing the WT MIC of the antibiotic by the mutant strain. Antibiotics tested: spectinomycin (SPC), azithromycin (AZITH), clarithromycin (CLR), erythromycin (ERY), capreomycin (CAPREO), hygromycin B (HYG B), amikacin (AMK), florfenicol amine (FF-NH_2), and linezolid (LZD). The wild-type (WT) genotype is *M. abscessus* ATCC19977; all other strains are derived from this WT.

**Day 14 Minimum Inhibitory Concentration (μg mL^{-1})**									
M. abscessus strains	**WT**	**Δ*whiB7***		**WhiB7 A51T**		**Δ*eis2***		**Eis2 T258R**	
**Compound**	**MIC**	**MIC**	**FC vs WT**	**MIC**	**FC vs WT**	**MIC**	**FC vs WT**	**MIC**	**FC vs WT**
SPC	512	32-64	**8-16**	64	**8**	512	**1**	512	**1**
AZITH	>32	2-4	**16-32**	4-8	**8-16**	>32	**1**	>32	**1**
CLR	2	0.25	**8**	0.5	**4**	2	**1**	2	**1**
ERY	>32	4	**16**	8	**8**	>32	**1**	>32	**1**
CAPREO	512	8-16	**32-64**	8	**32**	8-16	**32-64**	8	**64**
HYG B	256	8-16	**16-32**	16	**16**	8	**32**	16	**16**
AMK	4-8	0.25-0.5	**16-32**	0.5	**8-16**	0.25-0.5	**16-32**	0.25-0.5	**16-32**
FF-NH_2	62.5-125	250	**0.25-0.5**	250	**0.5**	1000	**0.125**	1000	**0.125**
LZD	8	8	**1**	8	**1**	8	**1**	8	**1**

Table S7| Florfenicol amine lacks mitochondrial protein synthesis inhibition activity and cytotoxicity. Table containing the compound name, mitochondrial protein synthesis (MPS) inhibitory activity, and cytotoxicity against human liver carcinoma cells (HepG2). MPS assay was conducted as described in ref [1]. Cytotoxicity was determined by 72-hour Cell-Titer Glo assay. Compounds tested: chloramphenicol (CAM), chloramphenicol amine (CAM-NH_2), florfenicol (FF), florfenicol acetyl (FF-ac), florfenicol amine (FF-NH_2), thiamphenicol (TAM), thiamphenicol amine (TAM-NH_2), linezolid (LZD), nitrofurantoin, saponin, and thioridazine. ND, not determined. IC_{50} and error (s.d.) are calculated from *n* = 3 biological replicates.

**Compound**	**HepG2 MPS IC_{50} (μM)**	**HepG2 Cytotoxicity IC_{50} (μM)**
CAM	5.5 ± 2.5	>200
CAM-NH_2	>100	>200
FF	1.2 ± 0.2	>200
FF-ac	4.7 ± 1.7	>200
FF-NH_2	> 100	>200
TAM	1.4 ± 0.7	>200
TAM-NH_2	>100	>200
LZD	6.2 ± 1.6	>200
Nitrofurantoin	ND	6.8 ± 0.3
Saponin	ND	12.6 ± 0.4
Thioridazine	ND	6.6 ± 0.3

Table S8| Metabolic stability of florfenicol amine. The metabolic stability half-lives and hepatocyte intrinsic clearance values for florfenicol amine (FF-NH_2) and the positive controls, verapamil and 7-hydroxycoumarin, in cryopreserved hepatocytes of the investigated three species, human, mouse and rat. The intrinsic clearance (CL_{int}) values for the positive control compounds were consistent with historical data and support the metabolic activity of the hepatocyte preparations used in this assay. Data shown as mean ± standard error; *n*=3.

Species	Compound	CL_{int} [µL/min/10^6 cells]	t½ [min]
Human	FF-NH_2	<0.53	>440
	Verapamil	5.22 ± 0.10	44.3 ± 0.8
	7-hydroxycoumarin	4.89 ± 0.01	47.3 ± 0.1
Rat	FF-NH_2	1.32 ± 0.09	131.7 ± 9.0
	Verapamil	12.9 ± 0.1	13.4 ± 0.1
	7-hydroxycoumarin	16.4 ± 0.9	10.6 ± 0.6
Mouse	FF-NH_2	2.51 ± 0.12	92.3 ± 4.4
	Verapamil	15.9 ± 0.5	14.6 ± 0.4
	7-hydroxycoumarin	38.0 ± 1.7	6.1 ± 0.3

Table S9| Summary of metabolites of florfenicol amine detected in hepatocyte stability assay samples. The fragmentation profiles from the Q-TOF mass spectrometry analysis, detailing the detected metabolites of florfenicol amine ($FF\text{-}NH_2$).

**Compound**	**Metabolic reaction**	**Molecular mass $[M+H]^+$**	**Hepatocyte species**		
			**Human**	**Rat**	**Mouse**
Parent	-	248.0751	✓	✓	✓
M1	Glucuronidation	424.1072	x	✓	✓
M2	Reduction	250.0908	x	x	✓
M3	Oxidative deamination	249.0591	x	x	x
M4	N-Acetylation	290.0857	x	x	x

✓ metabolite detected; x metabolite not detected

Table S10| Pharmacokinetic parameters for florfenicol amine in BALB/c mice after different routes of administration. Pharmacokinetic parameters for florfenicol amine (FF-NH_2) were obtained from plasma concentration-time courses. The terms C_{max} and t_{max} represent the maximum concentration achieved and the time to maximum concentration, respectively. The area under the concentration-time profile curve (AUC_{inf}) was calculated by the log-linear trapezoidal rule and was extrapolated to infinity by addition of the value C_{last}/λ_Z, where C_{last} is the concentration at the last sampling time point, and λ_Z the terminal slope of the log concentration-time curve determined by linear regression. Terminal half-life t½ is 0.693 divided by λ_Z. Absolute bioavailability F for the oral (PO) and subcutaneous (SC) administration were determined by calculating the dose normalized ratios of AUC_{inf} after administration relative to intravenous (IV) administration. Data shown for *n*=3/group.

**Dose**	**t_{max} [h]**	**C_{max} [mg L^{-1}]**	**AUC_{inf} [mg h L^{-1}]**	**t½ [h]**	**F [%]**
10 mg/kg IV	0.083	6.28	1.92	0.28	100
30 mg/kg PO	0.25	5.96	2.55	0.74	44
300 mg/kg PO	0.25	57.7	38.0	0.74	66
400 mg/kg SC	0.083	108	65.9	1.02	86

Table S11| List of genetically engineered *M. abscessus* and *M. smegmatis* strains and primers used.

Strain	Reference
M. smegmatis mc² 155	Snapper et al., 1990[2]
M. smegmatis pOLYG-*aac(3)IV*	This study
M. smegmatis pOLYG-*aac(3)IV-eis2*	This study
M. smegmatis pOLYG-*aac(3)IV-cat*	This study
M. abscessus ATCC 19977	Ripoll et al., 2009[3]
M. abscessus Δ2989 (*cat*)	This study
M. abscessus Δcat::cat complemented	This study
M. abscessus Δ3508c (*ΔwhiB7*)	Selchow et al., 2022[4]
M. abscessus ΔwhiB7 + pOLYG-*aac(3)IV*	This study
M. abscessus ΔwhiB7 + pOLYG-*aac(3)IV-eis2*	This study
M. abscessus Δ4532c (Δeis2)	Rominski et al., 2017[5]
M. abscessus Δeis2 + pOLYG-*aac(3)IV*	This study
M. abscessus Δeis2 + pOLYG-*aac(3)IV-eis2*	This study
M. abscessus WhiB7 A51T + pOLYG-*aac(3)IV*	This study
M. abscessus WhiB7 A51T + pOLYG-*aac(3)IV-eis2*	This study
M. abscessus Eis2 T258R + pOLYG-*aac(3)IV*	This study
M. abscessus Eis2 T258R + pOLYG-*aac(3)IV-eis2*	This study

List of primers

Name	Target	Primers	Amplicon (bp)
P1-up-cat fwd	flanking regions of *cat*	cacttcgcaatggccaagacgcggacgtgacagcgctg	1500
P2-up-cat rev		cccggacccagtgttcggcgggcacgcg	
P3-down-cat fwd		cgccgaacactgggtccgggactagtcg	1500
P4-down-cat-rev		ggcgtctgctaggacccgatcatgaccggaggcatgtg	
P5-comp-cat-fwd	single-copy expression of *cat*	aacgcgtgcggccgcggtacattatggctcagccgtgac	1221
P6-comp-cat-rev		tctagaggatccccgggtacctagtcccggacccaatc	
P7-probe-cat-fwd	SB probe of *cat*	ctcagccgtgacgaattgg	328
P8-probe-cat-rev		aaatctgttcagagagcgcc	
P11-exp-eis2-fwd	multi-copy expression of *eis2*	cgaggtcgacggtatcgatatcgcgacaactagaagtc	1372
P-12-exp- eis2 -rev		actgcaggaattcgatatcacggtccacacagttgttc	
P-13-exp-cat-fwd	Multi-copy expression of cat	cgaggtcgacggtatcgataCTGCTGGGACTGGGACGG	840
P-14-exp-cat-fwd		actgcaggaattcgatatcaCTAGTCCCGGACCCAATCC	
P-15-whiB7-san-fwd	Sanger sequencing of *whiB7* mutants	gatggggagcaatggttat	400
P-16-whiB7-san-fwd		gataagacaccgatacgag	
P-17-eis2-san-fwd	Sanger sequencing of *eis2* mutants	tgctgttgttcctgttcat	1482
P-18-eis2-san-fwd		ccacacagttgttcctttc	
P19- pOLYG-aac(3)IV-fwd	Colony PCR screening of multi-copy expression vector (pOLYG-*aac(3)IV*)	tggcttgtccaagggtgtat	1587(*eis2*) / 1095 (*cat*)
P20- pOLYG-aac(3)IV-rev		cgagatccgttgatcttcct	

Chemistry Materials and Methods

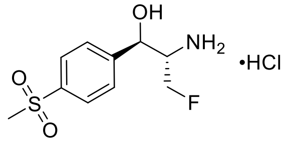

(1*R*,2*S*)-2-amino-3-fluoro-1-(4-(methylsulfonyl)phenyl)propan-1-ol hydrochloride (**Florfenicol amine**) 2,2-dichloro-N-((1*R*,2*S*)-3-fluoro-1-hydroxy-1-(4-(methylsulfonyl)phenyl)propan-2-yl)acetamide (20.0 g, 55.8 mmol) was suspended in water (500 mL) in a 1L round-bottom flask. Concentrated HCl (15.0 mL) was added to the solution, and the resulting reaction mixture was heated to reflux for three days. The solvent was removed under reduced pressure to provide the crude residue. Toluene (2x15 mL) was added to the residual material and evaporated under reduced pressure. The resulting powder was dissolved in a small amount of methanol and recrystallized by adding acetone dropwise. The precipitate was filtered and collected, yielding the product as a white solid (14.5 g, 51.1 mmol, 92%). ^{1}H NMR (500 MHz, D_2O) δ 8.04 (d, *J* = 8.0 Hz, 2H), 7.75 (d, *J* = 8.1 Hz, 2H), 5.10 (d, *J* = 8.3 Hz, 1H), 4.65 (ddd, *J* = 46.4, 11.4, 2.8 Hz, 1H), 4.43 (ddd, *J* = 46.3, 11.3, 4.9 Hz, 1H), 3.83 (dp, *J* = 23.8, 3.4 Hz, 1H), 3.28 (s, 3H). ^{13}C NMR (126 MHz, D_2O) δ 145.3, 139.3, 128.0 (2C), 127.8 (2C), 81.2, 79.9, 69.4, 56.4, 56.3, 43.2. MS (ESI): *m/z* 283.7$(M+H)^+$. Elemental analysis of the theoretical composition for empirical formula C10H14FNO3S·HCl·0.3 H2O: Calculated percentages are C, 41.54; H, 5.44; N, 4.84; F, 6.57; S, 11.09; Cl, 12.26. Found percentages are C, 41.72; H, 5.58; N, 4.69; F, 6.32; S, 10.87; Cl, 12.02.

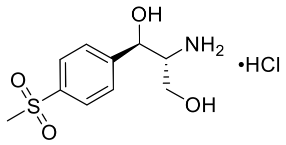

(1*R*,2*R*)-2-amino-1-(4-(methylsulfonyl)phenyl)propane-1,3-diol hydrochloride (**Thiamphenicol amine**). 2,2-dichloro-N-((1R,2R)-1,3-dihydroxy-1-(4-(methylsulfonyl)phenyl)propan-2-yl)acetamide (Thiamphenicol) (1.54 g, 4.21 mmol) was suspended in water within a 500 mL round-bottom flask containing 4 mL of concentrated hydrochloric acid. The resulting reaction mixture was heated under reflux for approximately 4 hours. After heating, the solvent was removed under reduced pressure. 15 mL of toluene was then added to the residue and evaporated to provide a dry residue containing (1R,2R)-2-amino-1-(4-nitrophenyl)propane-1,3-diol, HCl. The resulting residue was dissolved in a small aliquot of methanol. (1R,2R)-2-amino-1-(4-nitrophenyl)propane-1,3-diol, HCl was crystallized using acetone added

dropwise. Filtration of the solution resulted in pure (1R,2R)-2-amino-1-(4-(methylsulfonyl)phenyl)propane-1,3-diol, HCl (1.10 g, 3.90 mmol, 93 % yield) ^{1}H NMR (400 MHz, MeOD) δ 8.04 – 7.96 (m, 2H), 7.77 – 7.69 (m, 2H), 4.93 (d, J = 8.1 Hz, 1H), 3.63 (dd, J = 11.7, 3.7 Hz, 1H), 3.45 (dd, J = 11.7, 5.6 Hz, 1H), 3.35 (ddd, J = 8.7, 5.6, 3.7 Hz, 1H), 3.14 (s, 3H). ^{13}C NMR (151 MHz, MeOD) δ 148.4, 142.2, 129.0 (2C), 128.9 (2C), 71.2, 59.9, 59.7, 44.3. MS (ESI): *m/z* 246.3$(M+H)^{+}$.

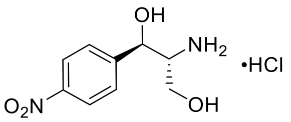

(1*R*,2*R*)-2-amino-1-(4-nitrophenyl)propane-1,3-diol hydrochloride (**Chloramphenicol amine**). 2,2-dichloro-N-((1R,2R)-1,3-dihydroxy-1-(4-nitrophenyl)propan-2-yl)acetamide (Chloramphenicol) (0.500 g, 1.55 mmol) was suspended in water inside a 500 mL round-bottom flask containing 4 mL of concentrated hydrochloric acid. The resulting reaction mixture was heated at reflux temperature for about 4 h. After heating, the solvent was removed under reduced pressure. Toluene (15 mL) was added to the resultant residue, and the toluene evaporated. Another 15 mL of toluene was then added to the residue and evaporated to provide a dry residue containing (1R,2R)-2-amino-1-(4-nitrophenyl)propane-1,3-diol, HCl. The resulting residue was dissolved in a small aliquot of methanol. (1R,2R)-2-amino-1-(4-nitrophenyl)propane-1,3-diol, HCl was crystallized using acetone added dropwise. Filtration of the solution resulted in pure (1R,2R)-2-amino-1-(4-nitrophenyl)propane-1,3-diol, HCl (0.325 g, 1.31 mmol, 84 % yield) ^{1}H NMR (400 MHz, MeOD) δ 8.29 – 8.21 (m, 2H), 7.69 – 7.61 (m, 2H), 4.81 (d, J = 5.5 Hz, 1H), 3.56 (dd, J = 10.8, 5.2 Hz, 1H), 3.42 (dd, J = 10.9, 5.9 Hz, 1H), 2.93 (q, J = 5.5 Hz, 1H). ^{13}C NMR (151 MHz, MeOD) δ 152.3, 148.7, 128.7 (2C), 124.6 (2C), 74.0, 64.0, 59.8. MS (ESI): *m/z* 242.2 $(M-H)^{-}$.

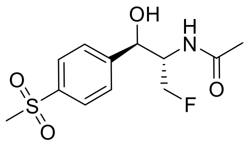

N-((1*R*,2*S*)-3-fluoro-1-hydroxy-1-(4-(methylsulfonyl)phenyl)propan-2-yl)acetamide (**Florfenicol acetyl**).

To a suspension of (1R,2S)-2-amino-3-fluoro-1-(4-(methylsulfonyl)phenyl)propan-1-ol hydrochloride (Florfenicol amine) (0.500 g, 1.76 mmol) in a 500 mL round-bottom flask with dry CH_2Cl_2 (60 mL), Et_3N (0.50 mL, 3.52 mmol) was added, and the mixture was stirred for 5 minutes. The homogeneous mixture was then cooled to 0°C. Acetyl chloride (0.152 g, 1.94 mmol) in dry CH_2Cl_2 (0.50 mL) was added dropwise, and the reaction mixture was allowed to warm to room temperature and stirred for 20 min. Diluted with water, washed with 1N HCl, saturated $NaHCO_3$, extracted with EtOAc (3x), dried (Na_2SO_4), and concentrated under reduced pressure to give the product as a white solid (0.260 g, 0.90 mmol, 51%). ^{1}H NMR (400 MHz, DMSO-d_6) δ 7.86 (m, 3H), 7.60 (d, *J* = 8.1 Hz, 2H), 5.96 (d, *J* = 4.6 Hz, 1H), 4.93 – 4.87 (m, 1H), 4.55 (ddd, *J* = 46.5, 8.3, 5.5 Hz, 1H), 4.35 – 4.17 (m, 2H), 3.19 (s, 3H), 1.74 (s, 3H). ^{13}C NMR (126 MHz, DMSO-d_6) δ 169.4, 148.6, 139.4, 127.2 (2 C), 126.4 (2 C), 82.8, 81.5, 69.6, 69.5, 53.7, 53.6, 43.5, 22.4. MS (ESI): *m/z* 290.4 $(M+H)^+$.

Source Data Inventory

Data	Location
Compound characterization	This document
Blots and other images	This document
Sequencing: Whole Genome Sequencing	This document<br>NCBI SRA accession PRJNA1141985
Sequencing: Transcriptomics	ExtendedData_RNAseq.xlsx<br>NCBI GEO accession GSE273574
Proteomics	ExtendedData_Proteomics.xlsx<br>proteomeXchange PXD059834<br>MassIVE MSV000096854
Accumulation	ExtendedData_Accumulation.xlsx
Drug combination studies	BRAID_FF-NH2_AnalysisReports.pdf
Main Figures	Source Data Figure 1.xlsx<br>Source Data Figure 2.xlsx<br>Source Data Figure 3.xlsx<br>Source Data Figure 5.xlsx
Extended Data Figures	Source Data Extended Data Figure 1.xlsx<br>Source Data Extended Data Figure 3.xlsx<br>Source Data Extended Data Figure 4 and Table S3.xlsx<br>Source Data Extended Data Figure 5.xlsx<br>Source Data Extended Data Figure 6.xlsx<br>Source Data Extended Data Figure 7.xlsx<br>Source Data Extended Data Figure 8.xlsx

Compound characterization

^1H NMR (D_2O,500 MHz)

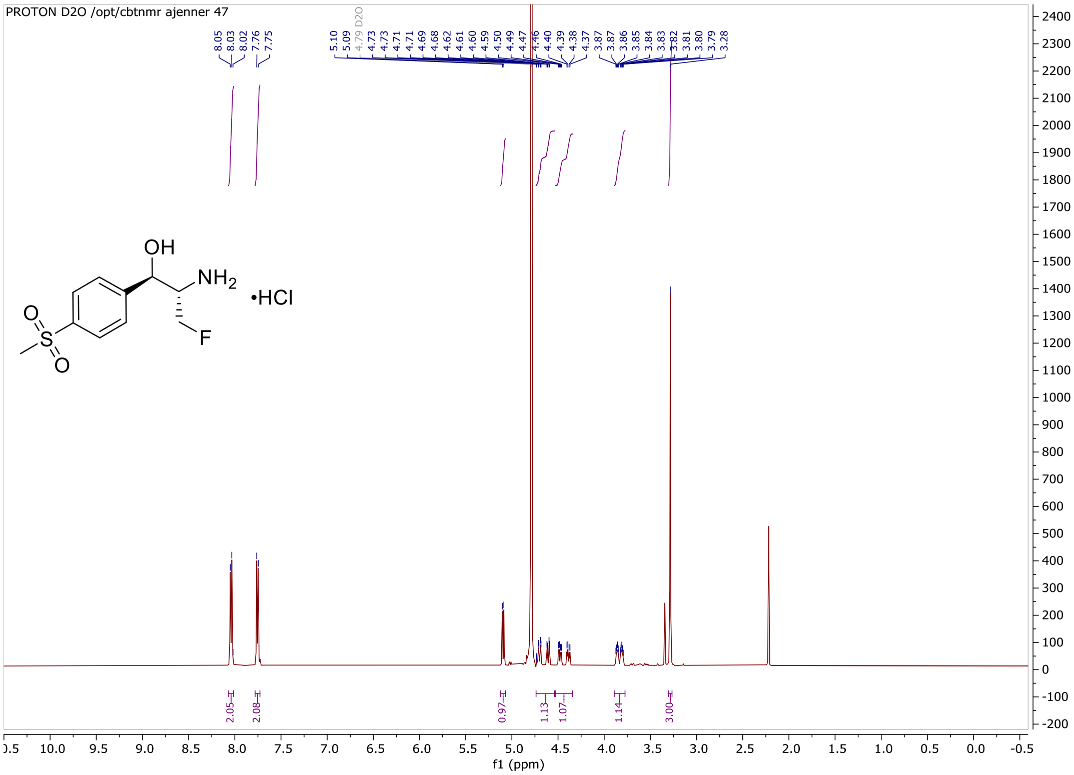

^{13}C NMR (D_2O, 126 MHz, D_2O)

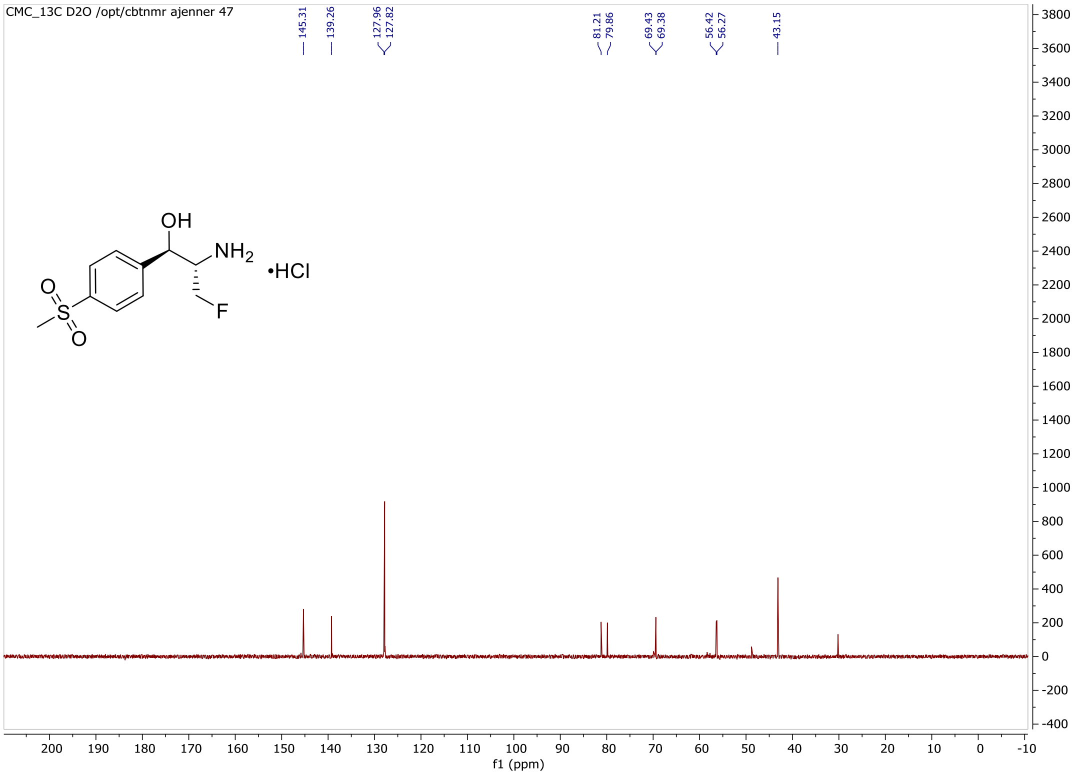

^{1}H NMR (400 MHz, MeOD)

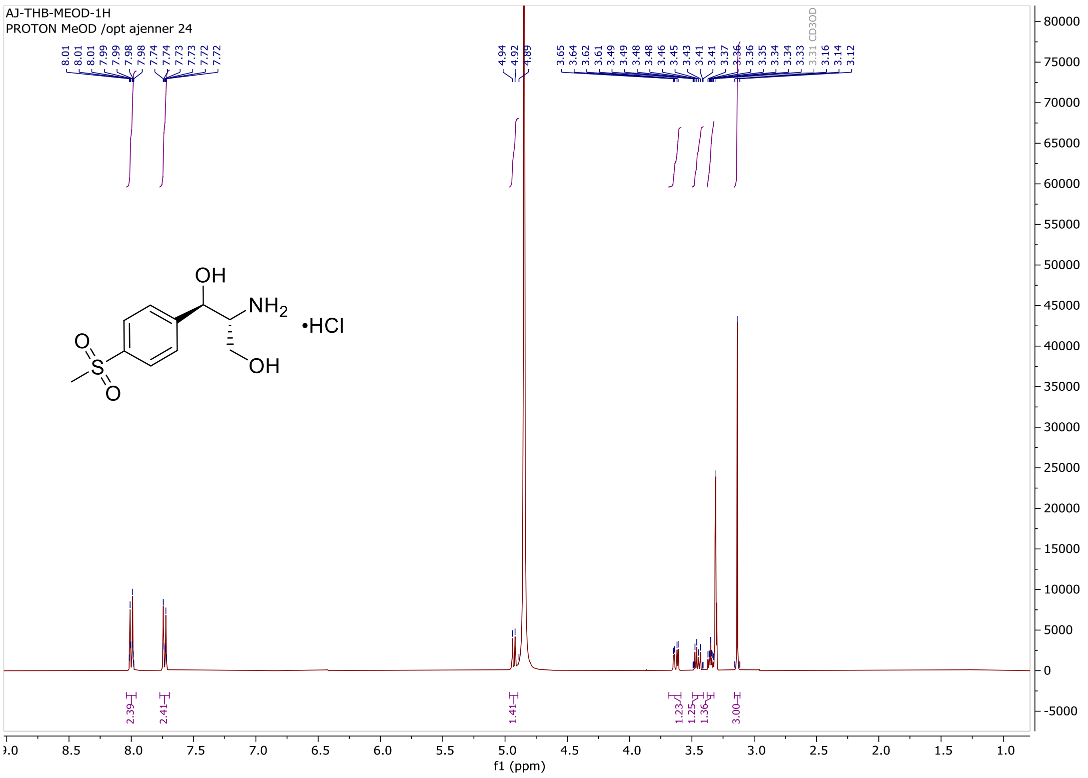

^{13}C NMR (151 MHz, MeOD)

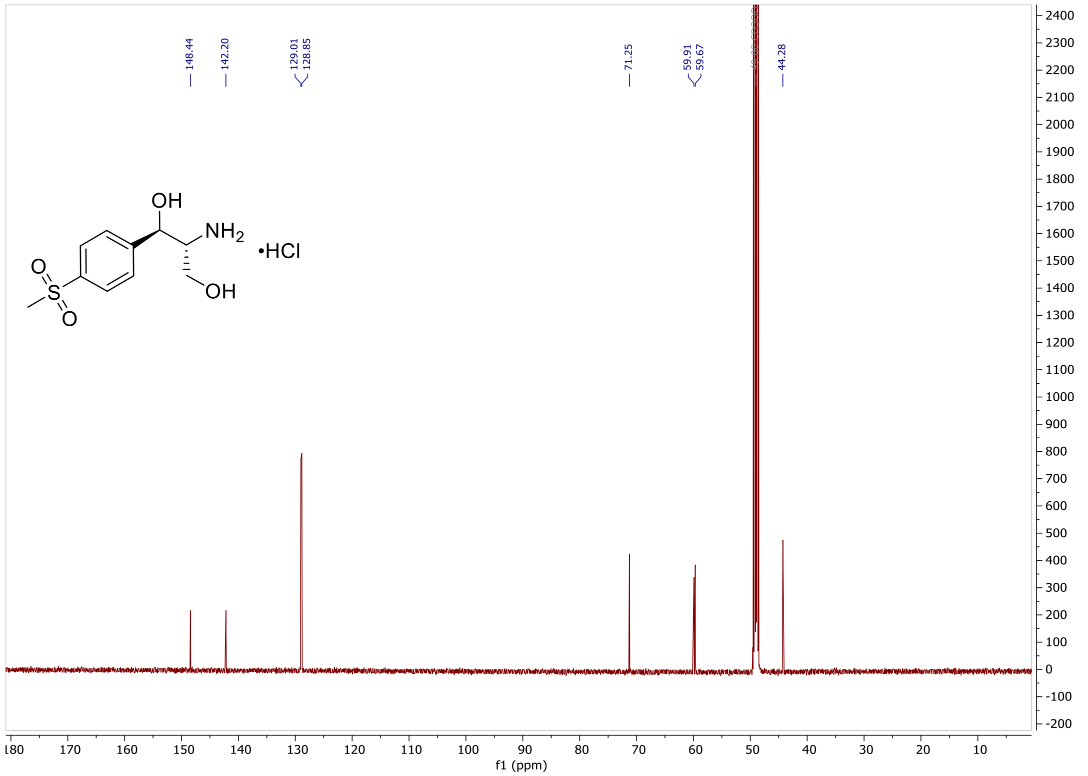

^{1}H NMR (400 MHz, MeOD)

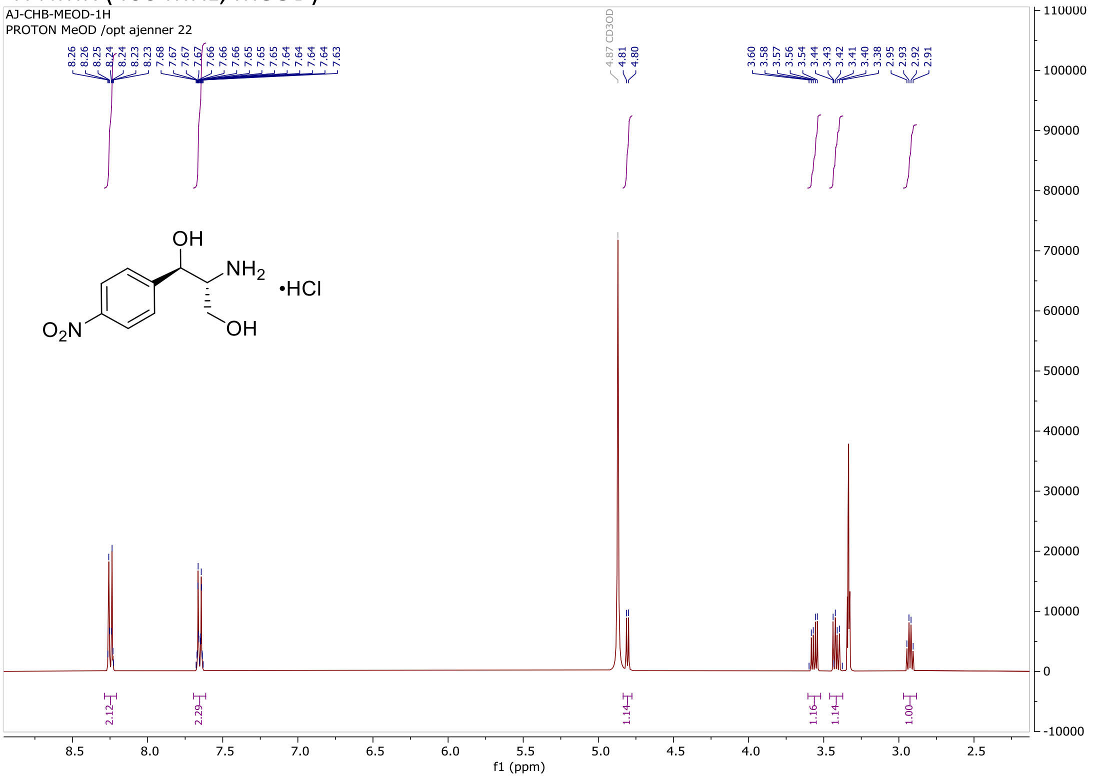

^{13}C NMR (151 MHz, MeOD)

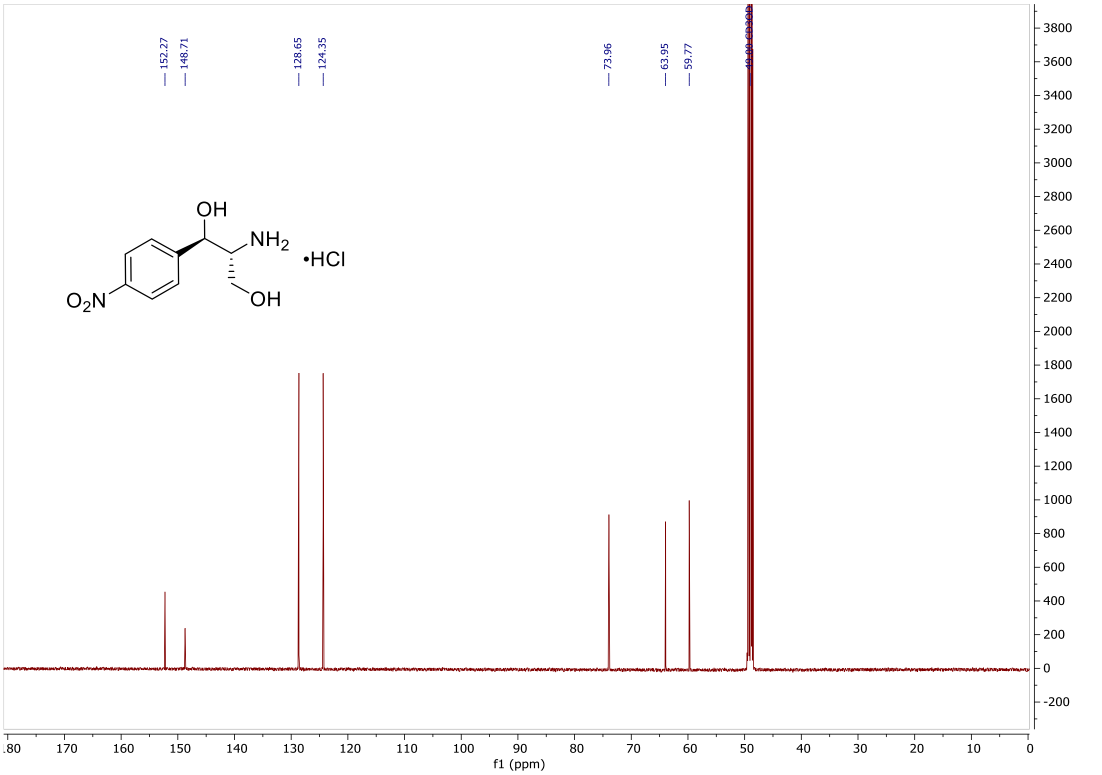

^{1}H NMR (DMSO-d_6, 400 MHz)

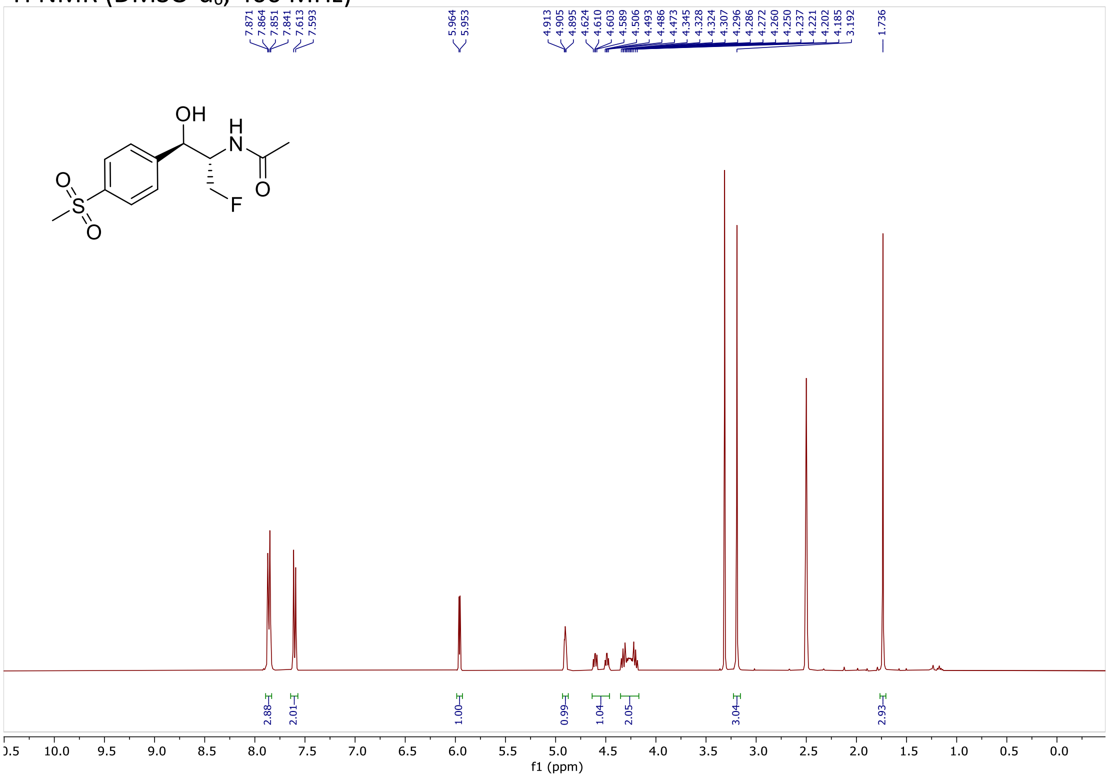

^{13}C NMR (DMSO-d_6. 126 MHz)

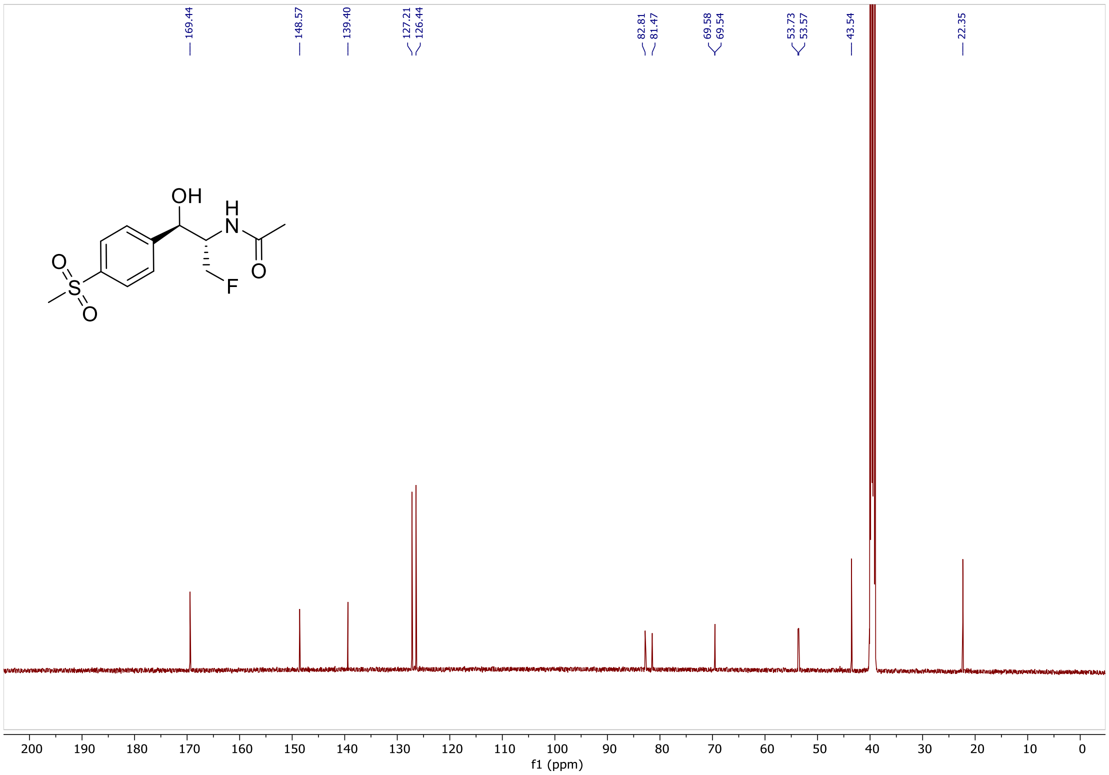

Source Data for Blots and Uncropped Images

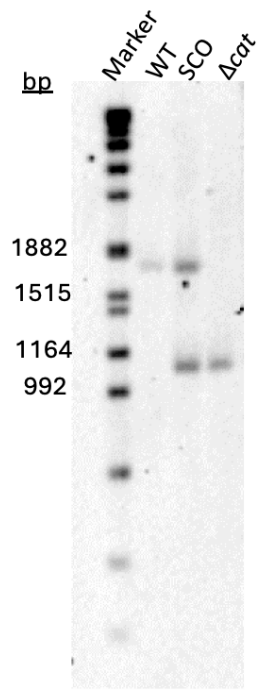

Lane	Expected bp
WT	1768
SCO	1768 & 1107
Δ*cat*	1107

Figure S1| Southern Blot analysis confirms the deletion of cat (MAB_2989) from the genome of M. ab*scessus*. Marker, Roche DNA Molecular Weight Marker VII; WT, *M. abscessus* ATCC 19977; SCO, 3' Single crossover; Δ, *MAB_2989* knock-out mutant).

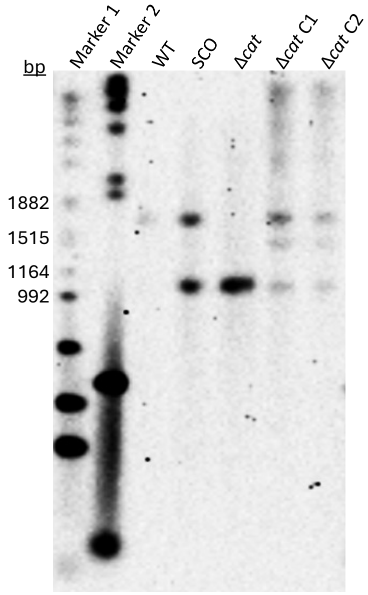

Lane	Expected bp
WT	1768
SCO	1768 & 1107
Δ*cat*	1107
Δ*cat* C1	1768 & 1107
Δ*cat* C2	1768 & 1107

Figure S2| Southern blot analysis of complementation of *cat* in *M. abscessus*. Marker 1, Roche DNA Molecular Weight Marker VII; Marker 2, Roche DNA Molecular Weight Marker II; WT, *M. abscessus* ATCC 19977; SCO, 3' Single crossover; Δ*cat*, *M. abscessus* ATCC 19977 *cat* (*MAB_2989)* knock-out mutant; Δ*cat* C1/C2, *M. abscessus cat* complement clones 1 and 2.

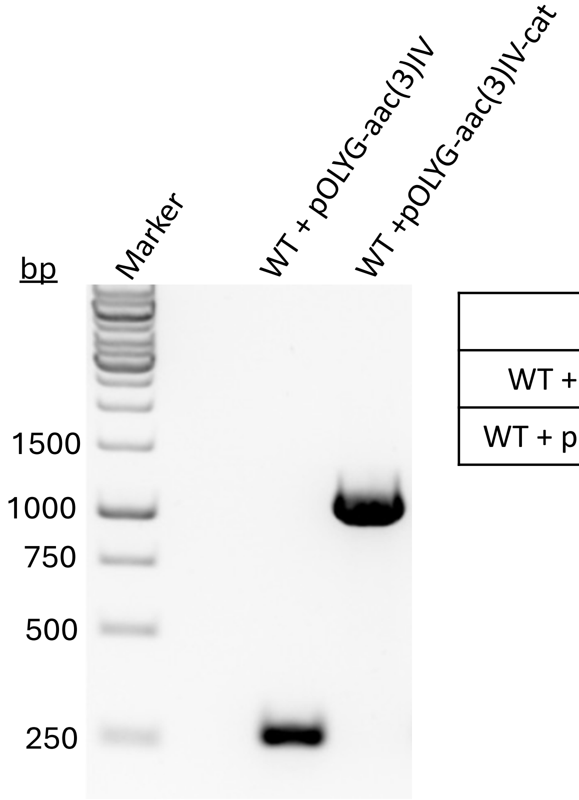

Lane	Expected bp
WT + pOLYG-aac(3)IV	259
WT + pOLYG-aac(3)IV-cat	1095

Figure S3| Gel Image of colony PCR of recombinant *M. smegmatis* pOLYG-*aac(3)IV-cat*. Marker, Generuler 1kb ladder; WT + pOLYG-aac(3)IV, *M. smegmatis* mc2-155 harboring pOLYG-*aac(3)IV-empty* vector; WT + pOLYG-aac(3)IV-cat, *M. smegmatis* colony harboring pOLYG-*aac(3)IV-cat* vector.

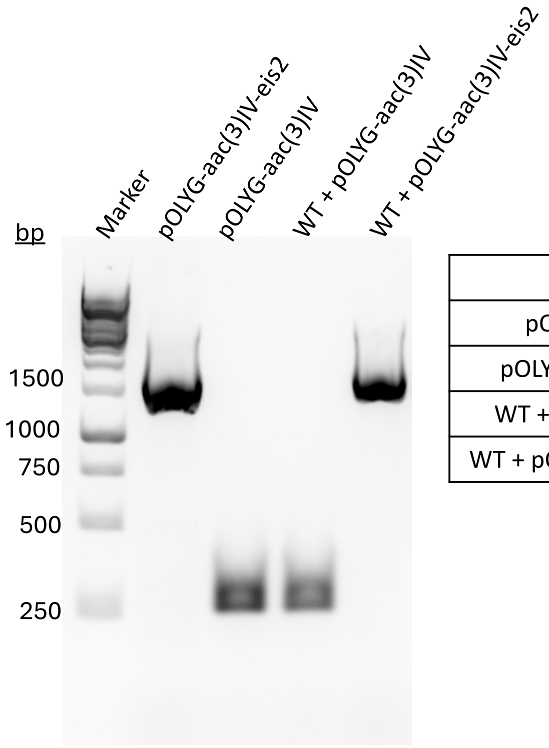

Lane	Expected bp
pOLYG-aac(3)IV	259
pOLYG-aac(3)IV-eis2	1587
WT + pOLYG-aac(3)IV	259
WT + pOLYG-aac(3)IV-eis2	1587

Figure S4| Gel Image of colony PCR of recombinant *M. smegmatis* pOLYG-*aac(3)IV-eis2*.

Marker, Generuler 1kb ladder; pOLYG-aac(3)IV-eis2, pOLYG-*aac(3)IV-eis2* vector only; pOLYG-aac(3)IV, pOLYG-*aac(3)IV*-backbone vector only; WT + pOLYG-aac(3)IV, *M. smegmatis* mc2-155 harboring pOLYG-*aac(3)IV*-backbone vector; WT + pOLYG-aac(3)IV-eis2, *M. smegmatis* mc2-155 harboring pOLYG-*aac(3)IV-eis2* vector.

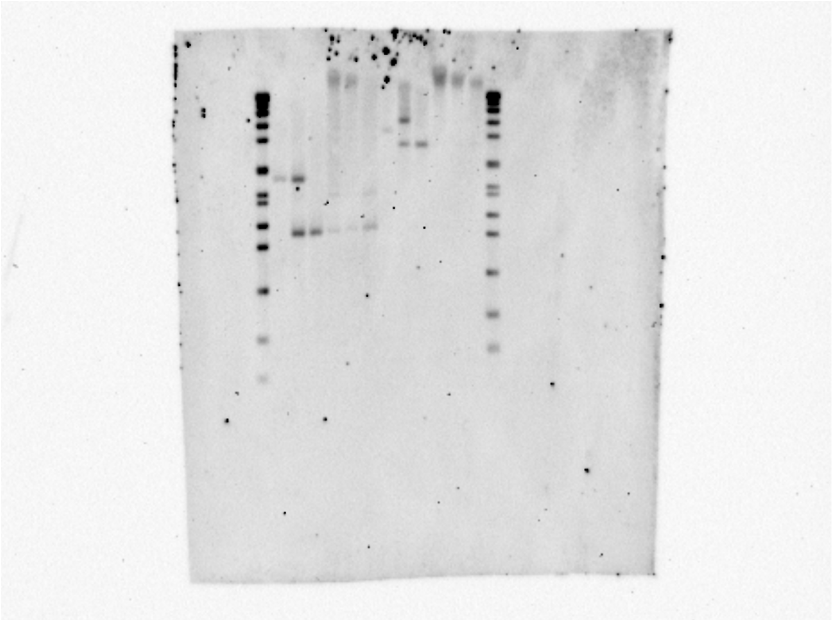

Original, uncropped gel image corresponding to Figure S1.

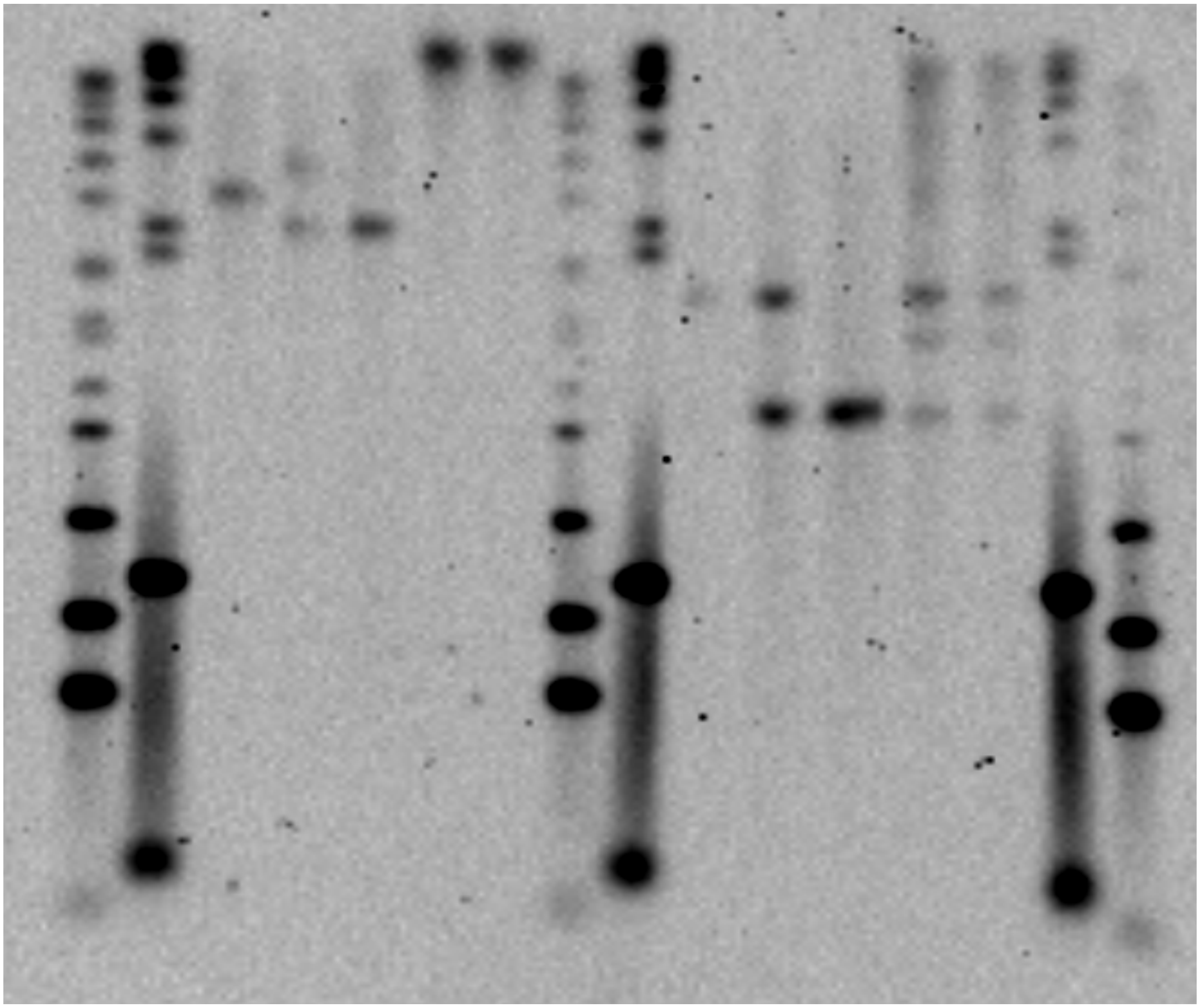

Original, uncropped gel image corresponding to Figure S2.

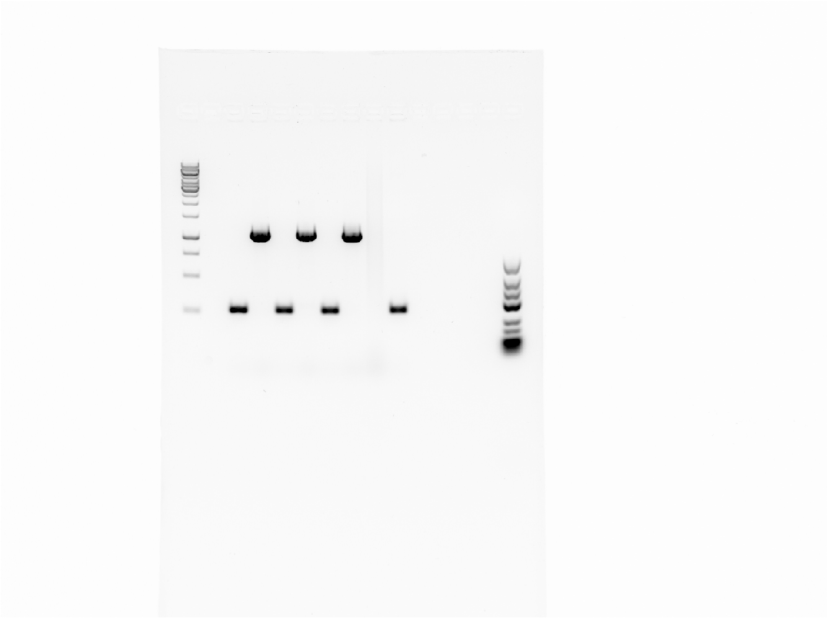

Original, uncropped gel image corresponding to Figure S3.

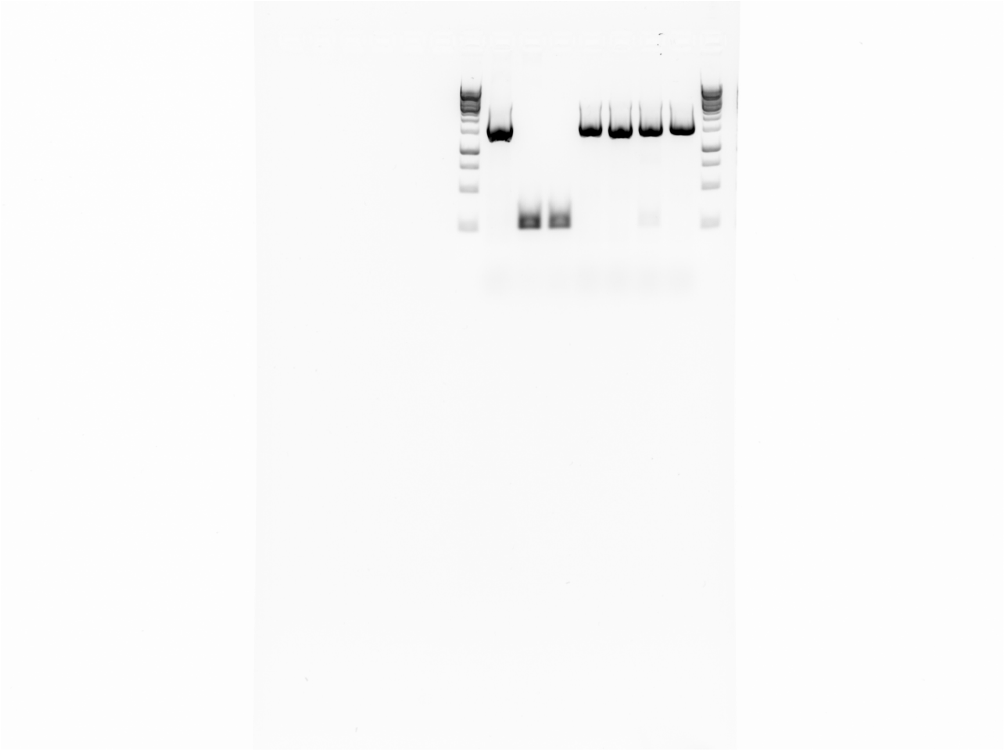

Original, uncropped gel image corresponding to Figure S4.

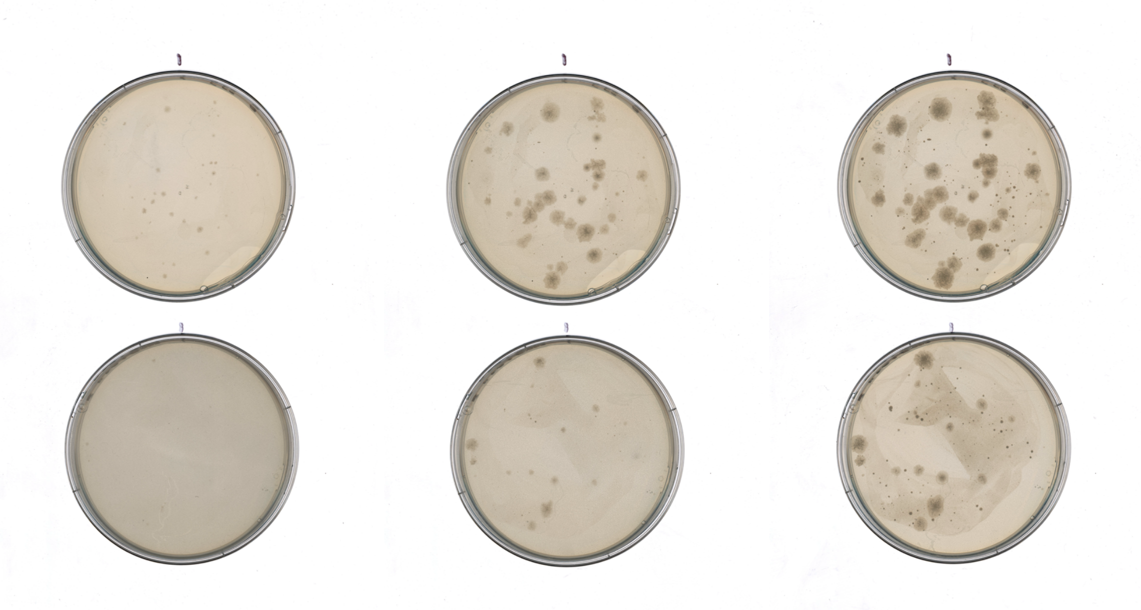

Original agar plate images corresponding to Extended Data Figure 2, panel a. Images from Day 6, Day 8, and Day 10 are presented from left to right. For each date, the top plate shows the image used in the main figure.

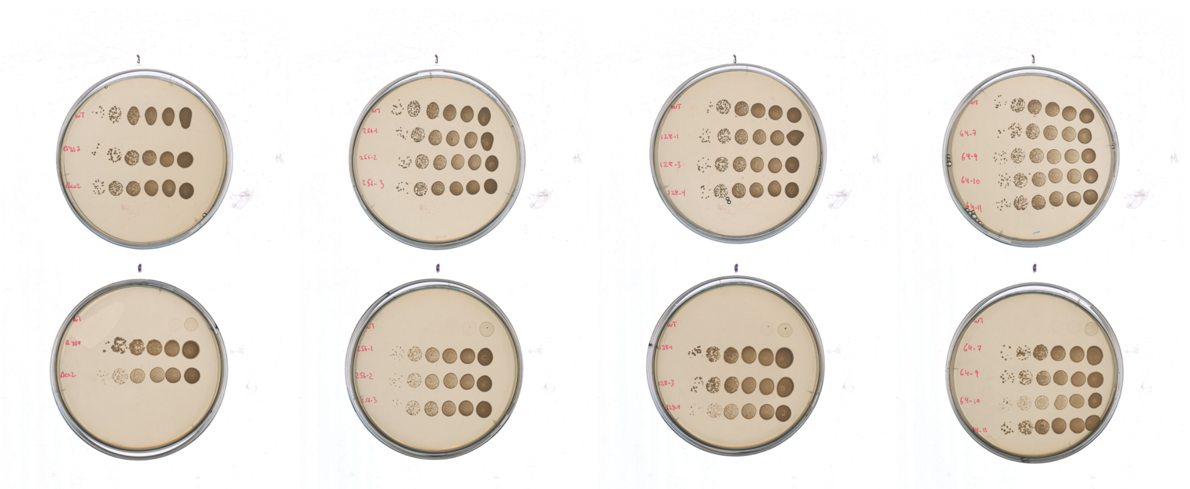

Original agar plate images corresponding to Extended Data Figure 2, panel b. In each image, the top agar plate is antibiotic-free, whereas the bottom plate contains 64 µg mL-1 of FF-NH_2.

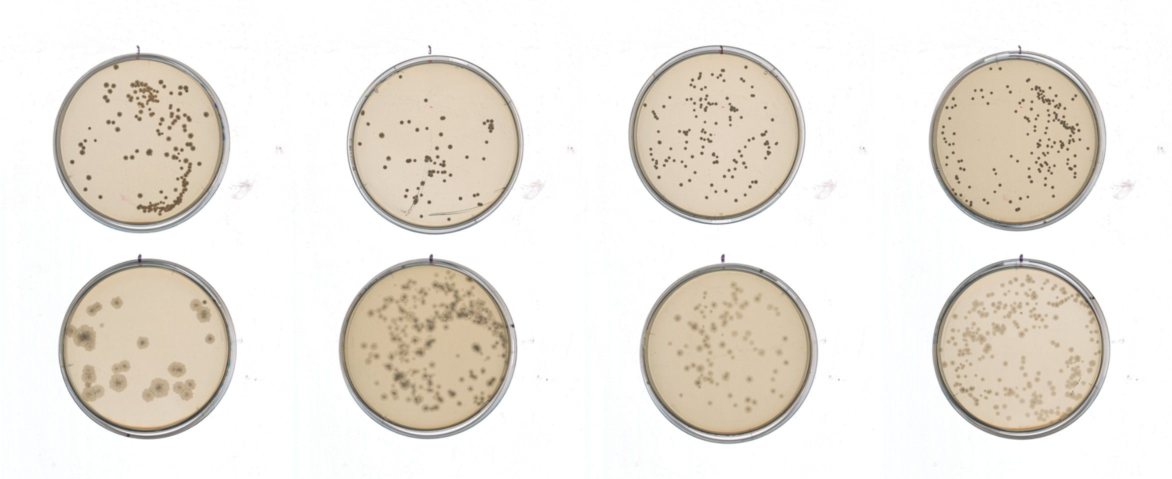

Original agar plate images corresponding to Extended Data Figure 2, panel c. In each image, the top agar plate is antibiotic-free, whereas the bottom plate contains 64 µg mL^{-1} of FF-NH_2**.** The two left images show *M. abscessus* Δ*whiB7* after the first (far left) and second (second from left) passages; the two right images show *M. abscessus* A51T after the first (second from right) and second (far right) passages.

Supplementary References

1 Boshoff, H. I. *et al.* Mtb-Selective 5-Aminomethyl Oxazolidinone Prodrugs: Robust Potency and Potential Liabilities. *ACS Infectious Diseases* **10**, 1679-1695 (2024).

2 Snapper, S., Melton, R., Mustafa, S., Kieser, T. & Jr, W. J. Isolation and characterization of efficient plasmid transformation mutants of Mycobacterium smegmatis. *Molecular microbiology* **4**, 1911-1919 (1990).

3 Ripoll, F. *et al.* Non mycobacterial virulence genes in the genome of the emerging pathogen Mycobacterium abscessus. *PloS one* **4**, e5660 (2009).

4 Selchow, P. *et al.* Apramycin overcomes the inherent lack of antimicrobial bactericidal activity in Mycobacterium abscessus. *Antimicrobial Agents and Chemotherapy* **66**, e01510-01521 (2022).

5 Rominski, A. *et al.* Elucidation of Mycobacterium abscessus aminoglycoside and capreomycin resistance by targeted deletion of three putative resistance genes. *Journal of Antimicrobial Chemotherapy* **72**, 2191-2200 (2017).
