# Supplementary material for: Prodrug florfenicol amine is activated by intrinsic resistance to target Mycobacterium abscessus
Source: Nat Microbiol. 2025 Oct 30;10(11):2875–91. doi: 10.1038/s41564-025-02147-9 (PMC12578646; doi:10.1038/s41564-025-02147-9)
Supplement: Supplementary file 2 — Reporting Summary [file 41564_2025_2147_MOESM2_ESM.pdf]

nature portfolio

Corresponding author(s): Richard E. Lee, Peter Sander

Last updated by author(s): Aug 28, 2025

Reporting Summary

Nature Portfolio wishes to improve the reproducibility of the work that we publish. This form provides structure for consistency and transparency in reporting. For further information on Nature Portfolio policies, see our Editorial Policies and the Editorial Policy Checklist.

Statistics

For all statistical analyses, confirm that the following items are present in the figure legend, table legend, main text, or Methods section.

n/a	Confirmed	
☐	☒	The exact sample size (n) for each experimental group/condition, given as a discrete number and unit of measurement
☐	☒	A statement on whether measurements were taken from distinct samples or whether the same sample was measured repeatedly
☐	☒	The statistical test(s) used AND whether they are one- or two-sided<br>*Only common tests should be described solely by name; describe more complex techniques in the Methods section.*
☒	☐	A description of all covariates tested
☐	☒	A description of any assumptions or corrections, such as tests of normality and adjustment for multiple comparisons
☐	☒	A full description of the statistical parameters including central tendency (e.g. means) or other basic estimates (e.g. regression coefficient) AND variation (e.g. standard deviation) or associated estimates of uncertainty (e.g. confidence intervals)
☐	☒	For null hypothesis testing, the test statistic (e.g. F, t, r) with confidence intervals, effect sizes, degrees of freedom and P value noted<br>*Give P values as exact values whenever suitable.*
☒	☐	For Bayesian analysis, information on the choice of priors and Markov chain Monte Carlo settings
☒	☐	For hierarchical and complex designs, identification of the appropriate level for tests and full reporting of outcomes
☒	☐	Estimates of effect sizes (e.g. Cohen's d, Pearson's r), indicating how they were calculated

Our web collection on statistics for biologists contains articles on many of the points above.

Software and code

Policy information about availability of computer code

Data collection

Dose-response activity data were obtained using a PHERAstar FS Multilabel Reader (BMG) for antibacterial activity measurements and enzymatic assays, and a Synergy H1 Plate Reader (BioTek) for the translation inhibition assay. Drug accumulation data were collected with the Echo MS system, managed and analyzed using Sciex OS-MQ Analytics Software (AB Sciex). Next-generation sequencing was performed on a NovaSeq X Plus system (Illumina).

Data analysis

Detailed methodologies are provided in the Methods section and are available upon request from the corresponding author. The following programs and versions were utilized in this study:
GraphPad Prism (version 10.2.0)
Microsoft Excel (365)
CLC Genomics Workbench (version 23.0.2)
R (version 4.1.0)
Sciex OS-MQ Analytics Software (version 3.3.10; version 2.1.6)
bcl-convert (version 4.2.4)
subread (version 2.0.1)
STAR (version 2.7.1a)
FastQC (version 0.11.5)
MultiQC (version 1.15)
tidyverse (version 2.0.0)
ggplot2 (version 3.4.4)
gplots (version 3.1.3 and 3.2.0)
edgeR (version 4.4.2)

limma (version 3.62.2)
magrittr (version 2.0.3)
NMF (version 0.23)
RColorBrewer (version 1.1-3)
JUMP shiny (jumpshiny.genenetwork.org)
braidrm (version 1.0.3)

For manuscripts utilizing custom algorithms or software that are central to the research but not yet described in published literature, software must be made available to editors and reviewers. We strongly encourage code deposition in a community repository (e.g. GitHub). See the Nature Portfolio guidelines for submitting code & software for further information.

Data

Policy information about availability of data

All manuscripts must include a data availability statement. This statement should provide the following information, where applicable:

- Accession codes, unique identifiers, or web links for publicly available datasets
- A description of any restrictions on data availability
- For clinical datasets or third party data, please ensure that the statement adheres to our policy

Whole-genome sequencing reads associated with this study have been submitted to the NCBI Sequence Read Archive (SRA) under accession number PRJNA1141985. Raw RNA-seq data reads have been deposited in the NCBI Gene Expression Omnibus (GEO) under accession number GSE273574. Raw mass spectra from proteomics experiment have been deposited to proteomeXchange and MassIVE repositories with identifiers PXD059834 and MSV000096854. All data supporting the findings of this study are provided within the Article, Supplementary Information (SI), Extended Datasets, and Source Data files.

Research involving human participants, their data, or biological material

Policy information about studies with human participants or human data. See also policy information about sex, gender (identity/presentation), and sexual orientation and race, ethnicity and racism.

Reporting on sex and gender	NA
Reporting on race, ethnicity, or other socially relevant groupings	NA
Population characteristics	NA
Recruitment	NA
Ethics oversight	NA

Note that full information on the approval of the study protocol must also be provided in the manuscript.

Field-specific reporting

Please select the one below that is the best fit for your research. If you are not sure, read the appropriate sections before making your selection.

☒ Life sciences ☐ Behavioural & social sciences ☐ Ecological, evolutionary & environmental sciences

For a reference copy of the document with all sections, see nature.com/documents/nr-reporting-summary-flat.pdf

Life sciences study design

All studies must disclose on these points even when the disclosure is negative.

Sample size	The drug dose-response assays presented in this manuscript are derived from a minimum of three independent experiments, except for the high-throughput susceptibility testing of the Mab mutants, which was conducted in duplicate per strain (13 strains tested total). Checkerboard assays were performed in triplicate, and MIC assays were conducted in at least biological duplicate. Accumulation experiments were performed in biological triplicate for those using Mab Δeis2 and Mab ΔwhiB7 strains, except for 0.167 h time point for Mab ΔwhiB7 (two replicates), and Mab WT strain was tested with a n=6. Transcriptomic and proteomic studies were conducted in biological triplicate. Hepatocyte stability studies were performed in triplicate. For the animal studies, pharmacokinetic studies were performed in triplicate and efficacy studies contained five animals per treatment group.
Data exclusions	No data were excluded
Replication	The frequency of each experiment is detailed in the figure legends and/or the corresponding Methods sections.
Randomization	Randomization was not considered for the in vitro experiments conducted in this study. GM-CSF mice (6-9 weeks old) were randomly assigned to treatment groups upon arrival.

Blinding	Blinding was not considered necessary for this study.

Reporting for specific materials, systems and methods

We require information from authors about some types of materials, experimental systems and methods used in many studies. Here, indicate whether each material, system or method listed is relevant to your study. If you are not sure if a list item applies to your research, read the appropriate section before selecting a response.

Materials & experimental systems

n/a	Involved in the study	
☐	☒	Antibodies
☐	☒	Eukaryotic cell lines
☒	☐	Palaeontology and archaeology
☐	☒	Animals and other organisms
☒	☐	Clinical data
☒	☐	Dual use research of concern
☒	☐	Plants

Methods

n/a	Involved in the study	
☒	☐	ChIP-seq
☒	☐	Flow cytometry
☒	☐	MRI-based neuroimaging

Antibodies

Antibodies used	The Ab110217 MitoBiogenesis™ In-Cell ELISA Kit (Abcam) was utilized to assess the drug-induced effects on mitochondrial biogenesis of the experimental and control compounds. Primary antibodies were supplied at a 200X concentration and secondary antibodies at a 2500X concentration. The experiment was conducted according to the manufacturer's instructions, with minor modifications pertaining to cell density as detailed in the Methods section.
Validation	Validation was conducted in-house by testing controls, including chloramphenicol (CAM). Our results demonstrated a similar propensity for CAM to inhibit mitochondrial protein synthesis (MPS), with an MPS IC50 of 5.5 +/- 2.5µM (Table S7), closely aligning with the manufacturer's reported value of 8.1 µM.

Eukaryotic cell lines

Policy information about cell lines and Sex and Gender in Research

Cell line source(s)	HepG2 cells were obtained from ATCC (ATCC HB-8065)
Authentication	Cell authentication was performed using STR-PCR.
Mycoplasma contamination	The cells tested negative for mycoplasma contamination.
Commonly misidentified lines (See ICLAC register)	No misidentified cell lines were used in this study.

Animals and other research organisms

Policy information about studies involving animals; ARRIVE guidelines recommended for reporting animal research, and Sex and Gender in Research

Laboratory animals	PK (UTHSC): Healthy male BALB/c mice (8-12 weeks old) weighing around 20 g were procured from Charles River (Wilmington, MA). All pharmacokinetic animal studies were conducted in accordance with the Animal Welfare Act and the Public Health Service Policy on Humane Care and Use of Laboratory Animals. Prior to initiation, the respective animal protocol was approved by the Institutional Animal Care and Use Committees of the University of Tennessee Health Science Center.<br><br>Efficacy (CSU): Female GM-CSF knockout mice (Strain #: 026812, B6.129S-Csf2tm1Mlg/J), aged 6–9 weeks, were purchased from the Jackson Laboratory. The mice were infected with M. abscessus via the intrapulmonary aerosol route in the Biosafety Level-3 (BSL-3) laboratory.
Wild animals	No wild animals were used in this study.
Reporting on sex	All animals were female.
Field-collected samples	No field-collected samples were used.
Ethics oversight	PK (UTHSC): All pharmacokinetic animal studies were conducted in accordance with the Animal Welfare Act and the Public Health Service Policy on Humane Care and Use of Laboratory Animals. Prior to initiation, the respective animal protocol (ACUC #24-0590) was approved by the Institutional Animal Care and Use Committees of the University of Tennessee Health Science Center.

Efficacy (CSU): All mouse experiments were approved by Colorado State University's Institutional Animal Care and Use Committee (IACUC), protocol #5157.

Note that full information on the approval of the study protocol must also be provided in the manuscript.

Plants

Seed stocks	NA
Novel plant genotypes	NA
Authentication	NA
