# Supplementary material for: Prodrug florfenicol amine is activated by intrinsic resistance to target Mycobacterium abscessus
Source: Nat Microbiol. 2025 Oct 30;10(11):2875–91. doi: 10.1038/s41564-025-02147-9 (PMC12578646; doi:10.1038/s41564-025-02147-9)

Combination Analysis

$FF\text{-}NH_2$ x anti-M. abscessus antibiotics

Note: FFA is synonymous with $FF\text{-}NH_2$ in this document

Analysis Run: July 25, 2024

braidrm version: 0.71

FFA vs. Lee2593 (AmSPC2593)

Observed Effect (Raw)

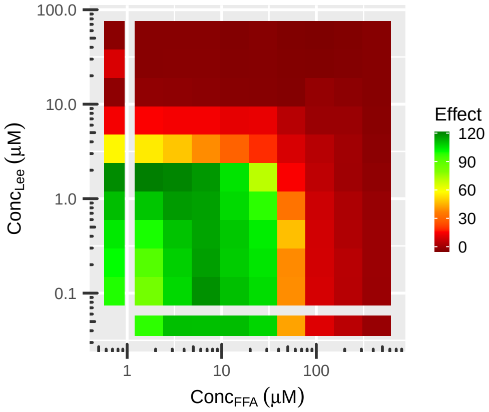

Best BRAID Fit

$ID_{M,A}$	48.5 (46.3 – 50.7)
$ID_{M,B}$	3.37 (3.26 – 3.47)
n_a	4.55 (4.03 – 5.37)
n_b	7.84 (6.28 – 9.89)
E_0	109.4428
E_f	0.6760932
κ	−0.284 (−0.36 – −0.204)

Additive Indices

IAE_{50}	1 (1 – 1)
IAE_{90}	1 (1 – 1)

BRAID Indices

IAE_{50}	1 (1 – 1)
IAE_{90}	1 (1 – 1)

Observed Effect

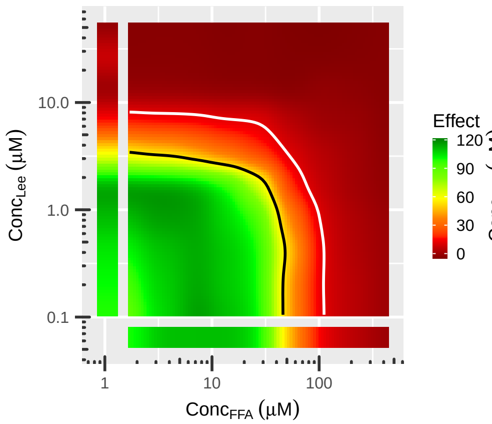

Additive Effect

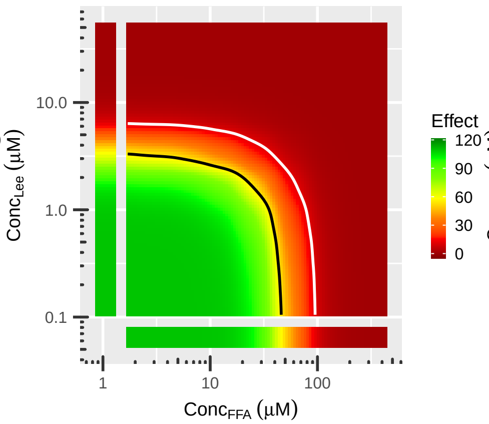

BRAID Effect

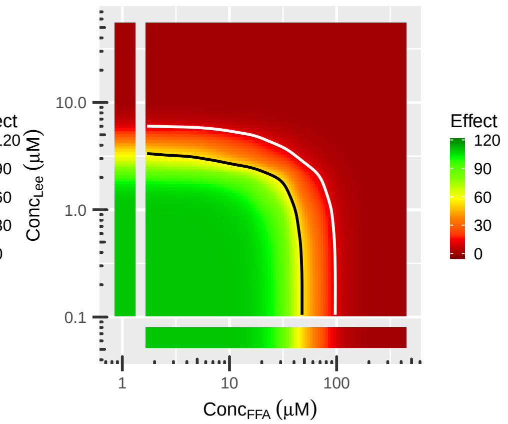

Interpolation Error

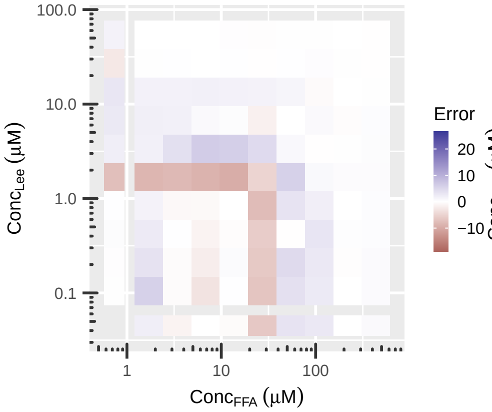

Additive Error ($R^2 = 0.987$)

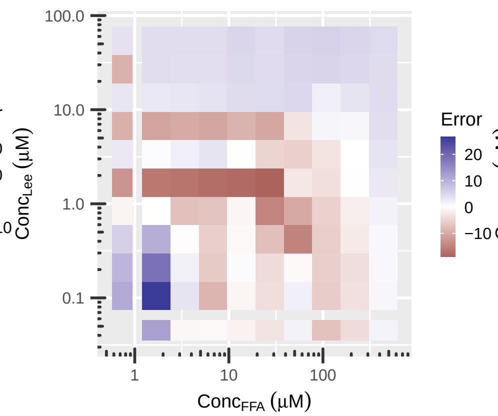

BRAID Error ($R^2 = 0.989$)

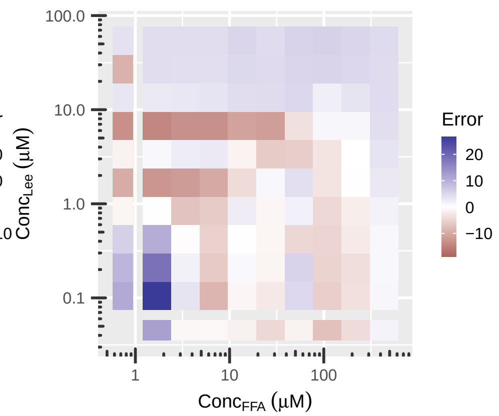

EC_{50} of FFA (μM)

Lee (μM)	Est
0	48.5 (46.3 – 50.7)
1	43.5 (41.7 – 45.4)
10	0 (0 – 0)
100	0 (0 – 0)

EC_{50} of Lee2593 (μM)

FFA (μM)	Est
0	3.37 (3.26 – 3.47)
10	2.95 (2.88 – 3.04)
100	0 (0 – 0)
1000	0 (0 – 0)

Potentiation of FFA

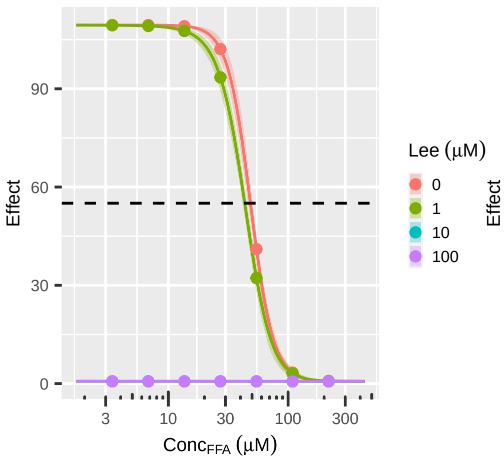

Potentiation of Lee2593

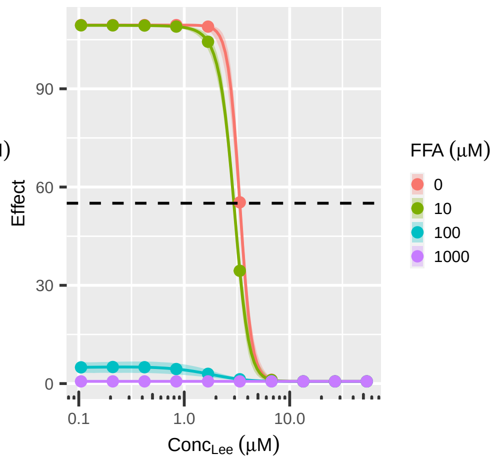

FFA vs. AMK

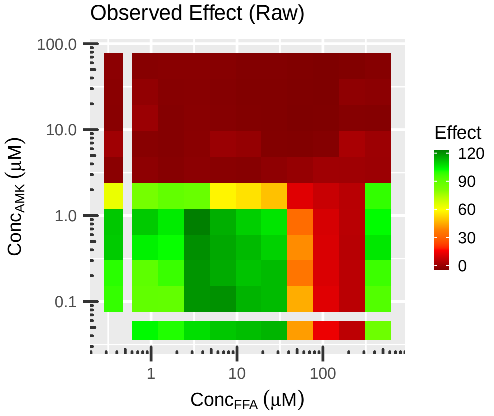

Best BRAID Fit

$ID_{M,A}$	36.3 (32.2 – 42.5)
$ID_{M,B}$	1.78 (1.67 – 1.95)
n_a	5.04 (3.86 – 7.27)
n_b	6.02 (4.32 – 8.61)
E_0	115.0739
$E_{f,A}$	26 (19.9 – 34.6)
$E_{f,B}$	−0.6011257
κ	−0.688 (−0.824 – −0.548)

Additive Indices

IAE_{50}	1 (1 – 1)
IAE_{90}	1 (1 – 1)

BRAID Indices

IAE_{50}	1 (1 – 1)
IAE_{90}	1 (1 – 1)

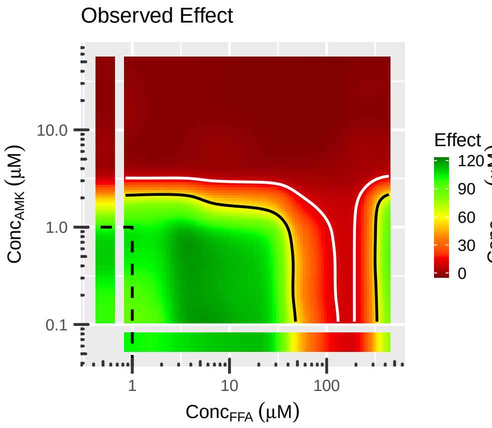

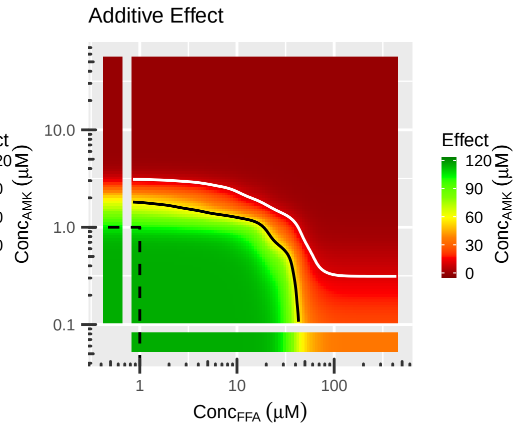

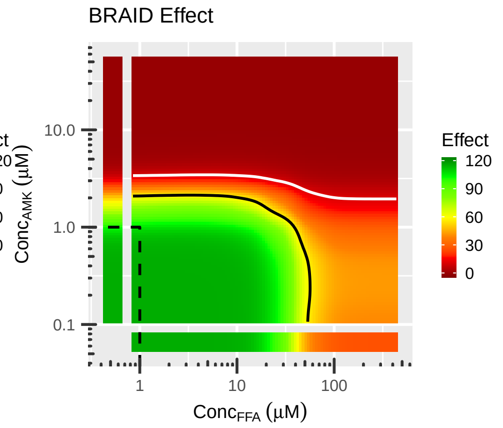

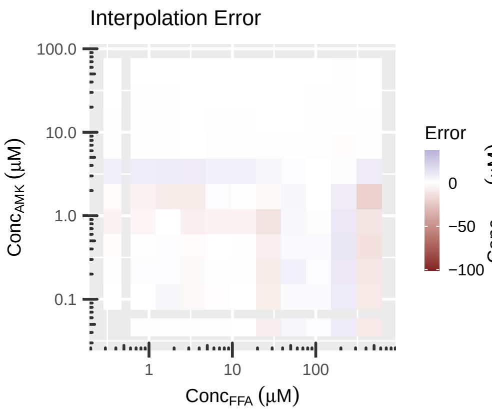

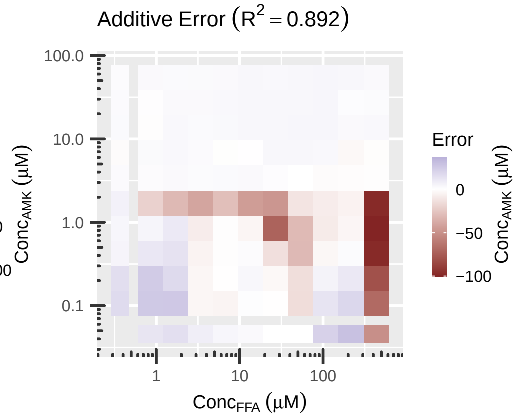

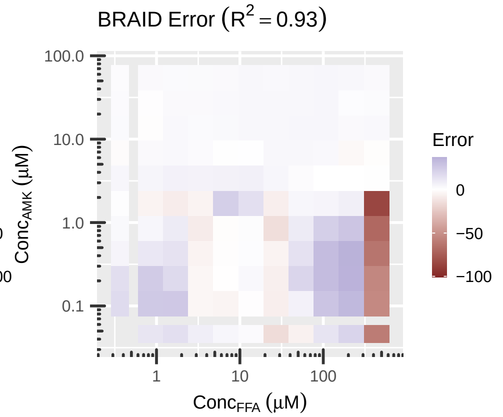

EC_{50} of FFA (μM)

AMK (μM)	Est
0	41 (35.7 – 49.4)
1	38.5 (34.1 – 46)
10	0 (0 – 0)
100	0 (0 – 0)

EC_{50} of AMK (μM)

FFA (μM)	Est
0	1.78 (1.67 – 1.95)
1	1.94 (1.85 – 2.11)
10	1.93 (1.81 – 2.17)
100	0 (0 – 0)

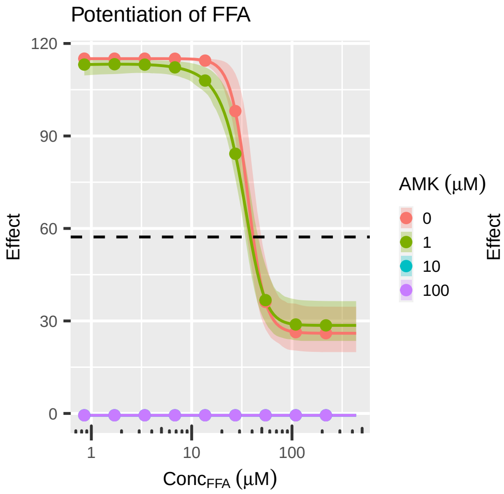

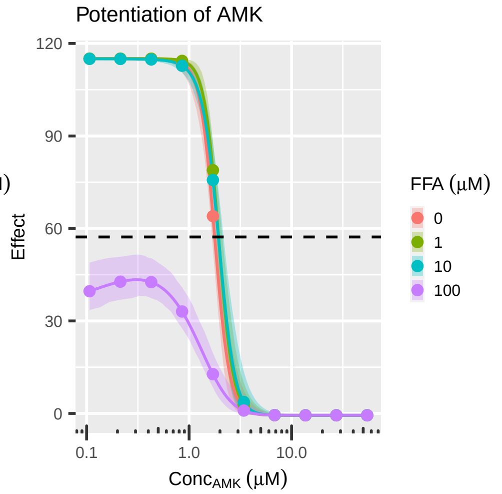

FFA vs. Azithromycin (AZITH)

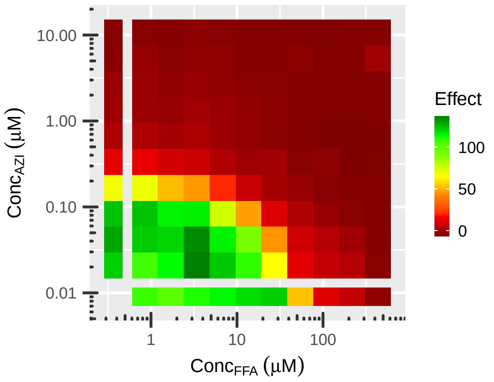

Observed Effect (Raw)

Best BRAID Fit	
$ID_{M,A}$	51.3 (48.6 – 53.7)
$ID_{M,B}$	0.201 (0.194 – 0.209)
n_a	5.14 (4.48 – 5.87)
n_b	3.93 (3.63 – 4.26)
E_0	120.5068
$E_{f,A}$	4.79 (1.96 – 8.23)
$E_{f,B}$	−3.018956
κ	1.23 (1.08 – 1.34)

Additive Indices	
IAE_{50}	2.33 (2.28 – 2.37)
IAE_{90}	1.72 (1.63 – 1.78)

BRAID Indices	
IAE_{50}	2.34 (2.31 – 2.37)
IAE_{90}	1.75 (1.71 – 1.8)

Observed Effect — Additive Effect — BRAID Effect

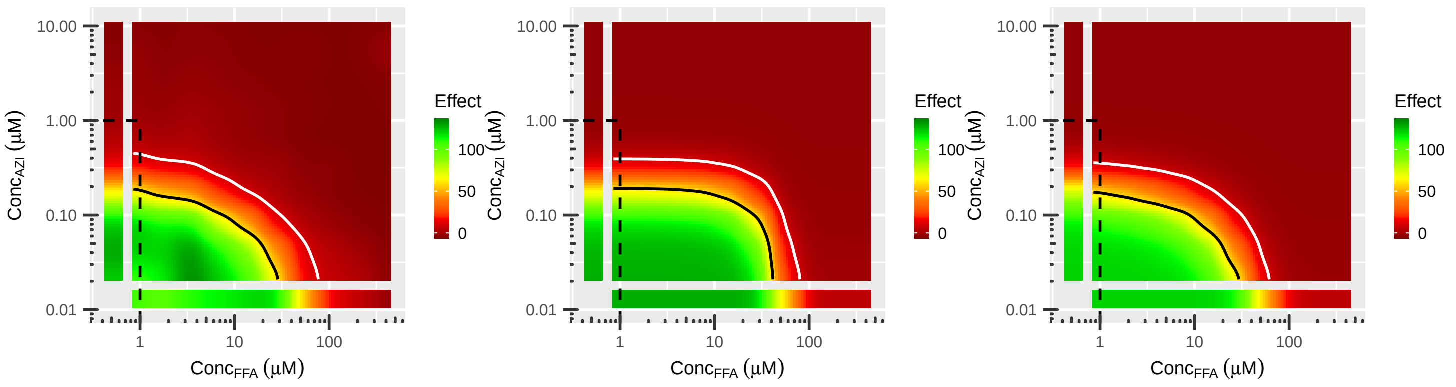

Interpolation Error — Additive Error $(R^2 = 0.966)$ — BRAID Error $(R^2 = 0.993)$

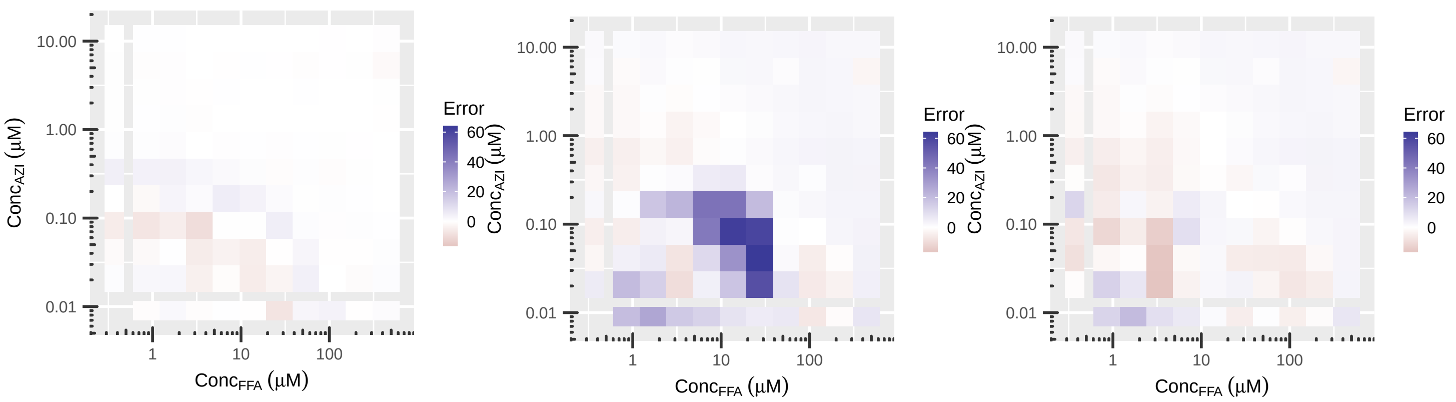

EC_{50} of FFA (μM)

AZI (μM)	Est
0	52.7 (50.1 – 54.9)
0.1	8.75 (8.31 – 9.38)
1	0 (0 – 0)
10	0 (0 – 0)

EC_{50} of AZITH (μM)

FFA (μM)	Est
0	0.201 (0.194 – 0.209)
1	0.171 (0.168 – 0.176)
10	0.0925 (0.0899 – 0.0963)
100	0 (0 – 0)

Potentiation of FFA — Potentiation of AZITH

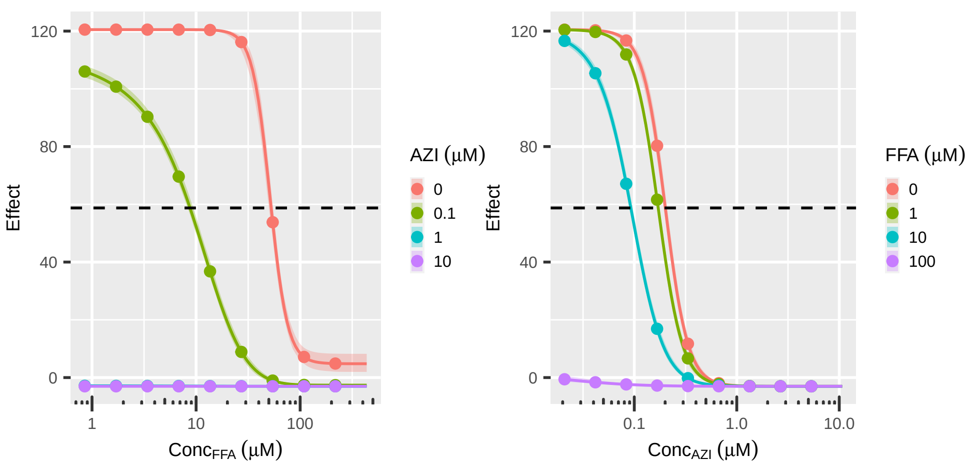

FFA vs. Cefoxitin (CEFOX)

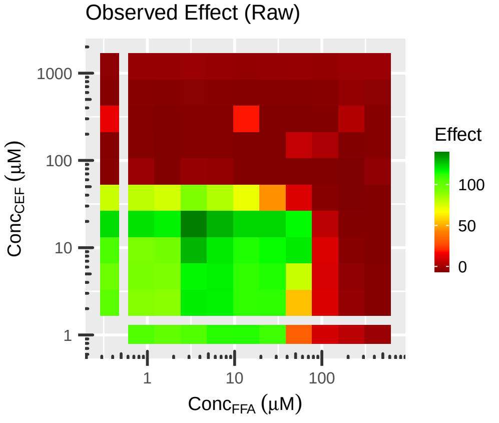

Best BRAID Fit

$ID_{M,A}$	54.9 (52 – 58.2)
$ID_{M,B}$	37.6 (36.5 – 38.9)
n_a	4.55 (3.83 – 5.63)
n_b	10 (7.68 – 10)
E_0	111.7263
E_f	– 3.521793
κ	– 0.814 (– 0.868 – – 0.726)

Additive Indices

IAE_{50}	1 (1 – 1)
IAE_{90}	1 (1 – 1)

BRAID Indices

IAE_{50}	1 (1 – 1)
IAE_{90}	1 (1 – 1)

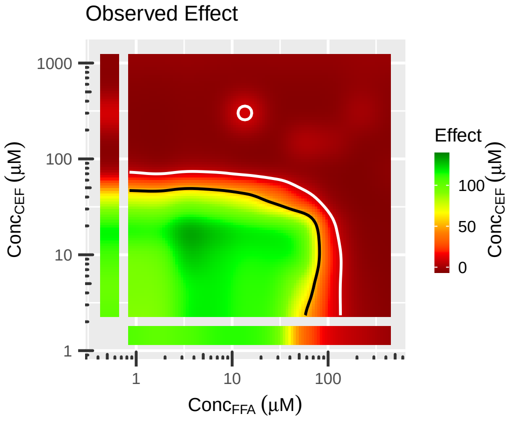

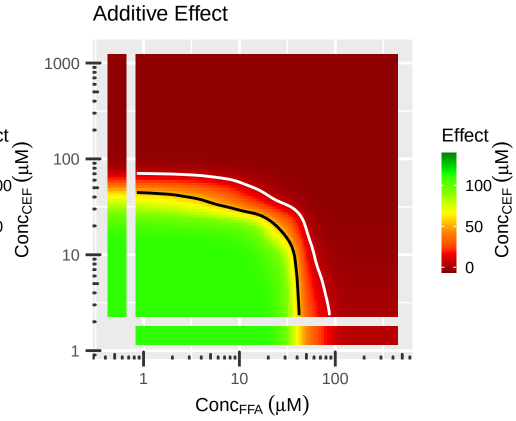

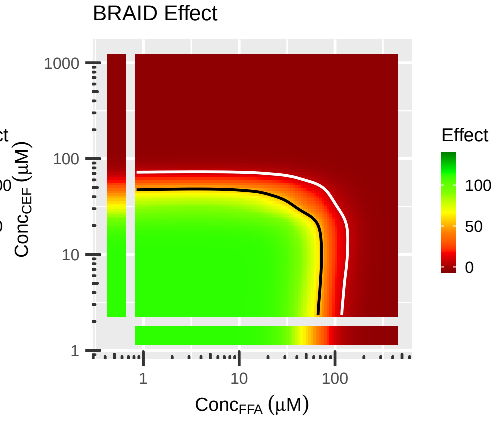

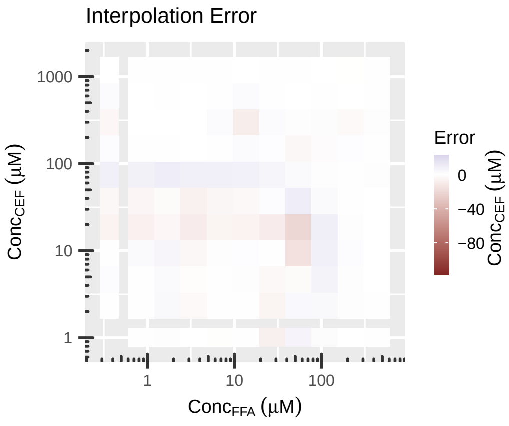

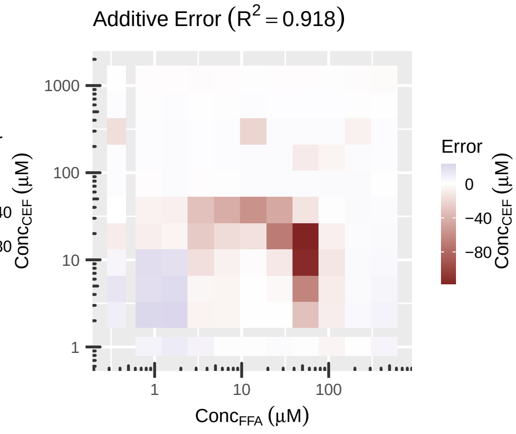

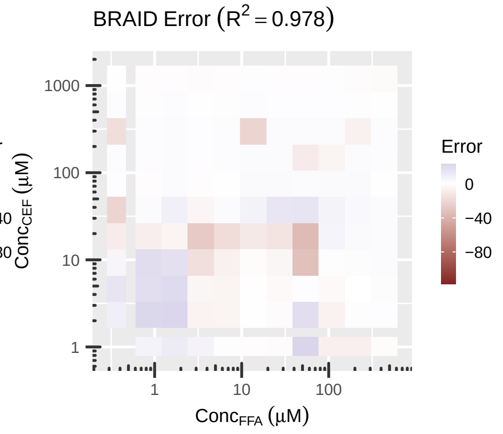

EC_{50} of FFA (μM)

CEF (μM)	Est
0	54.9 (52 – 58.2)
10	71.3 (67.5 – 74)
100	0 (0 – 0)
1000	0 (0 – 0)

EC_{50} of CEFOX (μM)

FFA (μM)	Est
0	37.6 (36.5 – 38.9)
1	41.6 (40.7 – 42.9)
10	42.1 (41.1 – 43.9)
100	0 (0 – 0)

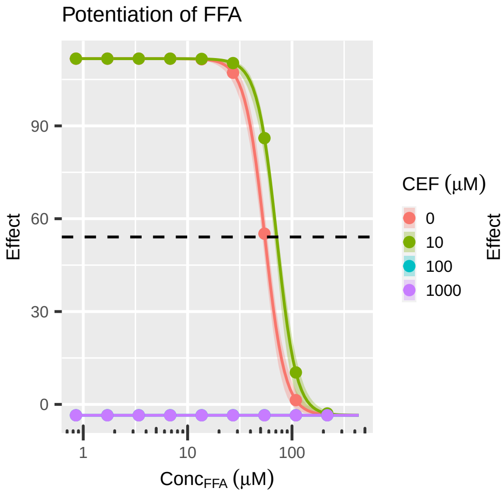

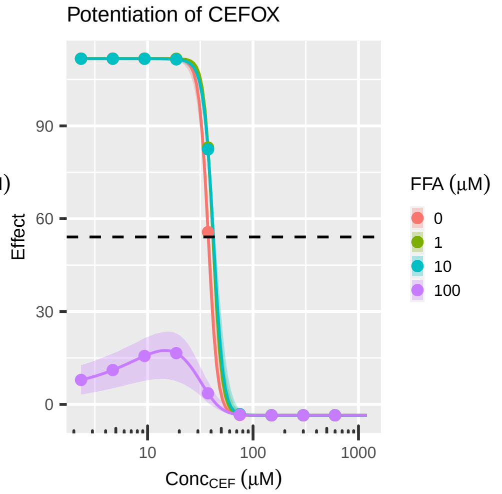

FFA vs. Clofazimine (CLO)

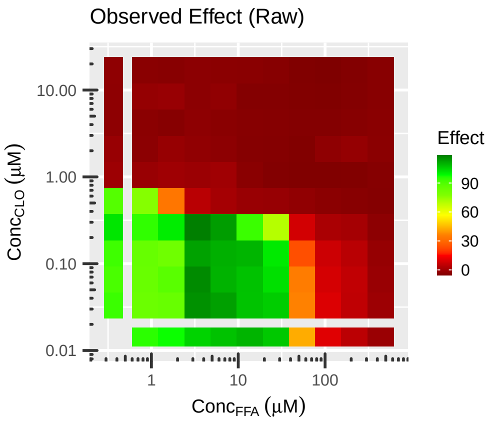

Best BRAID Fit

$ID_{M,A}$	53.9 (50.6 – 58.2)
$ID_{M,B}$	0.572 (0.55 – 0.595)
n_a	5.07 (4.19 – 6.51)
n_b	10 (7.95 – 10)
E_0	102.769
$E_{f,A}$	4.76 (1.16 – 9.74)
$E_{f,B}$	– 2.62457
κ	0.157 (0.0254 – 0.324)

Additive Indices

IAE_{50}	1.19 (1.12 – 1.24)
IAE_{90}	1.06 (1 – 1.11)

BRAID Indices

IAE_{50}	1.35 (1.33 – 1.37)
IAE_{90}	1.21 (1.16 – 1.22)

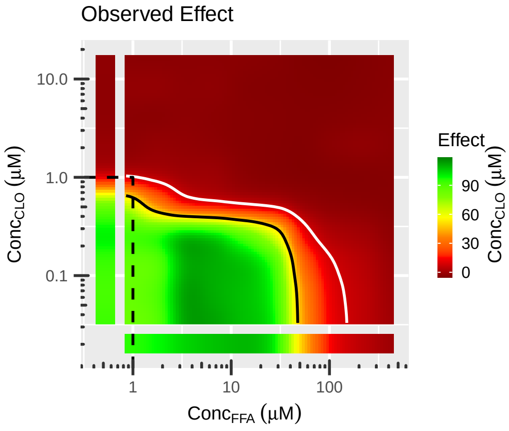

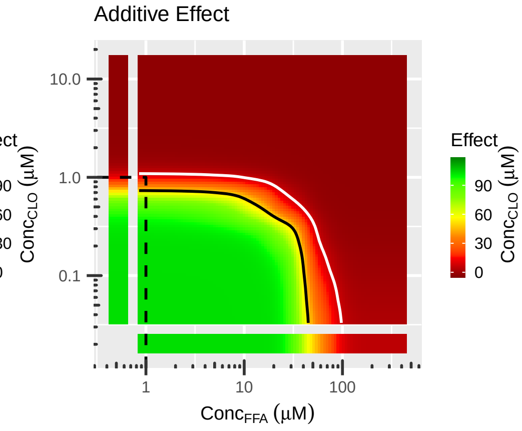

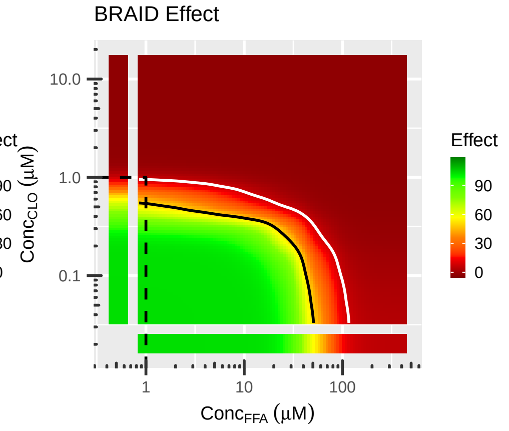

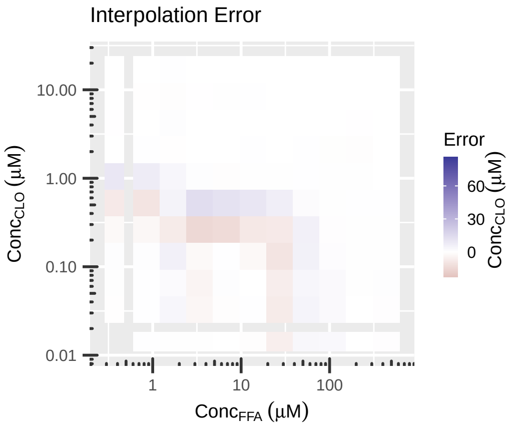

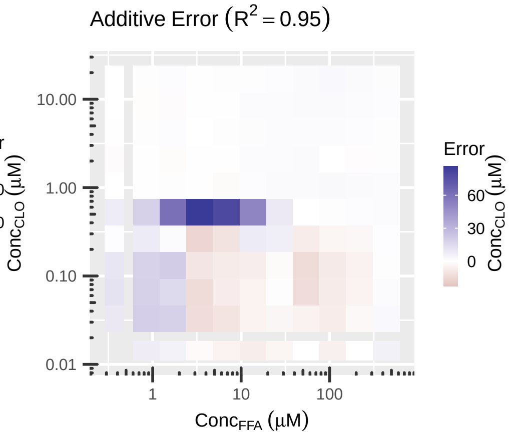

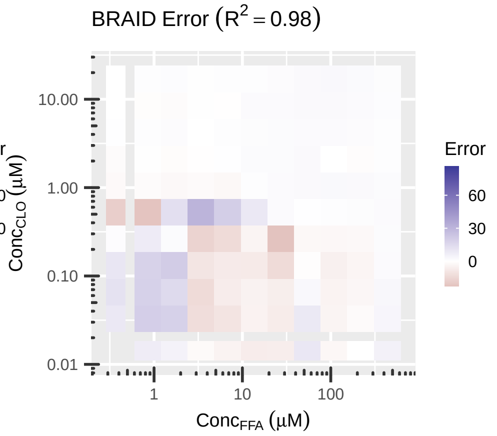

EC_{50} of FFA (μM)

CLO (μM)	Est
0	55.5 (52.9 – 59.8)
0.1	45.3 (43 – 47.5)
1	0 (0 – 0)
10	0 (0 – 0)

EC_{50} of CLO (μM)

FFA (μM)	Est
0	0.572 (0.55 – 0.595)
1	0.534 (0.522 – 0.546)
10	0.414 (0.401 – 0.43)
100	0 (0 – 0)

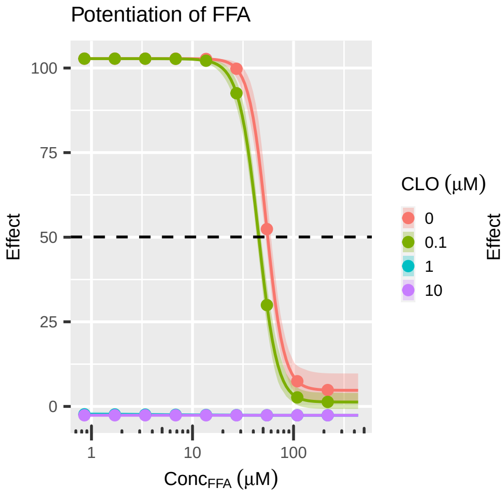

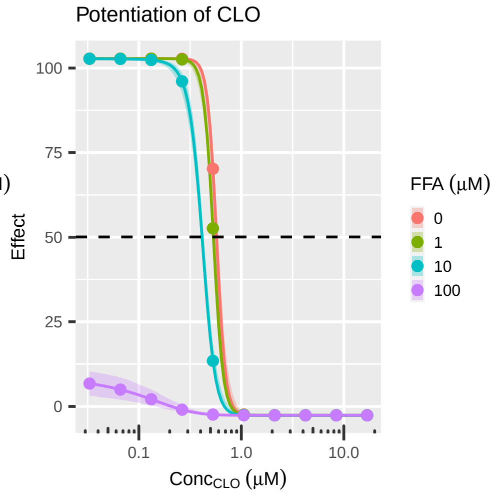

FFA vs. Clarithromycin (CLR)

Observed Effect (Raw)

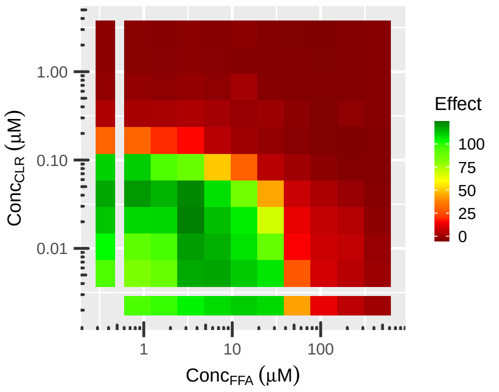

Best BRAID Fit

$ID_{M,A}$	50.4 (47.8 – 52.8)
$ID_{M,B}$	0.15 (0.143 – 0.158)
n_a	5.19 (4.62 – 5.91)
n_b	5.98 (5.29 – 6.78)
E_0	109.012
$E_{f,A}$	6.65 (4.03 – 10.8)
$E_{f,B}$	– 1.588071
κ	0.847 (0.694 – 0.981)

Additive Indices

IAE_{50}	2.58 (2.56 – 2.73)
IAE_{90}	2.31 (2.12 – 2.35)

BRAID Indices

IAE_{50}	2.71 (2.66 – 2.75)
IAE_{90}	2.23 (2.19 – 2.29)

Observed Effect

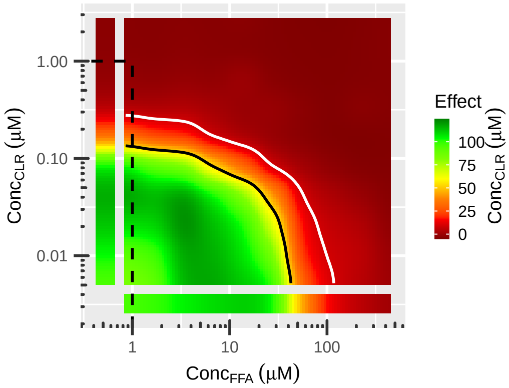

Additive Effect

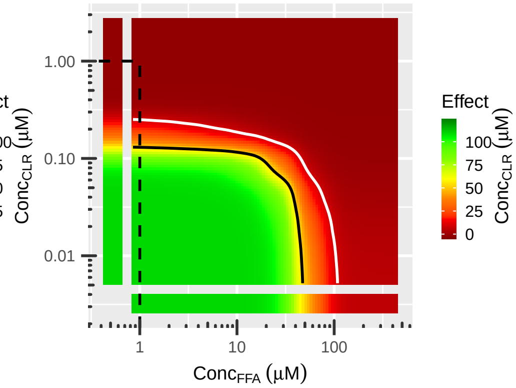

BRAID Effect

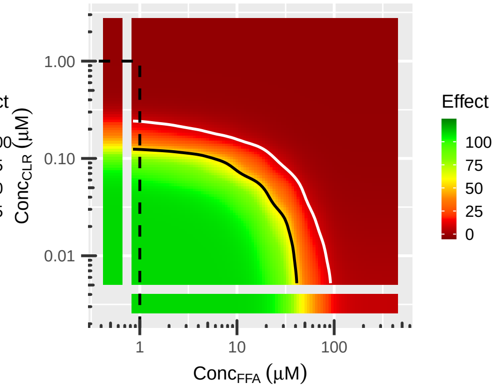

Interpolation Error

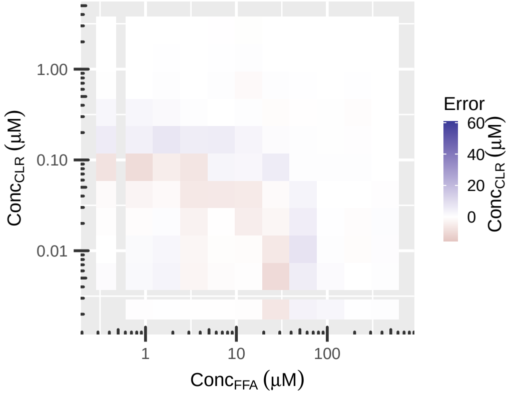

Additive Error ($R^2 = 0.972$)

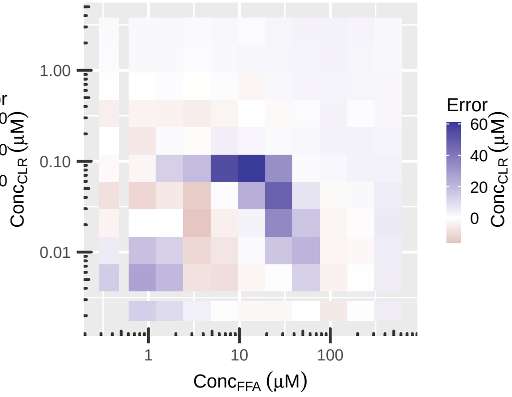

BRAID Error ($R^2 = 0.99$)

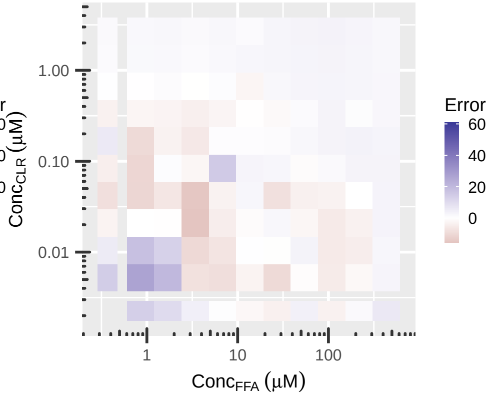

EC_{50} of FFA (µM)

CLR (µM)	Est
0	52 (49.3 – 54.5)
0.01	38.9 (37.9 – 40.3)
0.1	5.21 (4.77 – 5.94)
1	0 (0 – 0)

EC_{50} of CLR (µM)

FFA (µM)	Est
0	0.15 (0.143 – 0.158)
1	0.129 (0.126 – 0.133)
10	0.0789 (0.0767 – 0.0827)
100	0 (0 – 0)

Potentiation of FFA

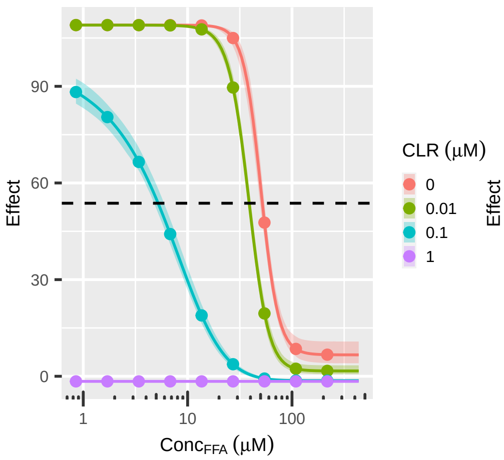

Potentiation of CLR

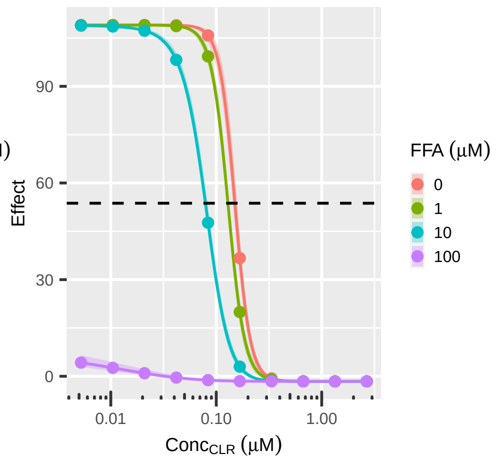

FFA vs. Eravacycline (ERAV)

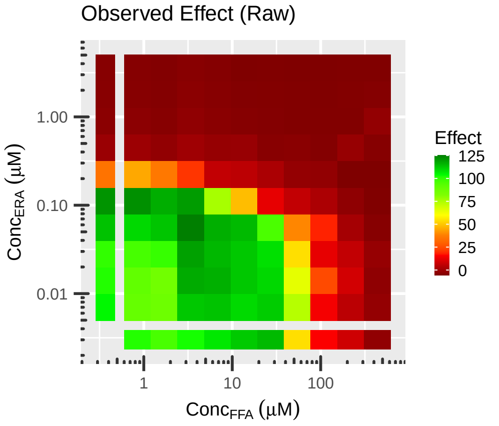

Best BRAID Fit

$ID_{M,A}$	70.5 (64.9 – 76.1)
$ID_{M,B}$	0.218 (0.207 – 0.229)
n_a	3.39 (2.97 – 3.88)
n_b	7.55 (6.17 – 9.25)
E_0	109.3566
E_f	– 2.658234
κ	0.554 (0.402 – 0.719)

Additive Indices

IAE_{50}	2.21 (2.18 – 2.32)
IAE_{90}	1.97 (1.78 – 2.01)

BRAID Indices

IAE_{50}	2.24 (2.21 – 2.28)
IAE_{90}	1.92 (1.87 – 1.97)

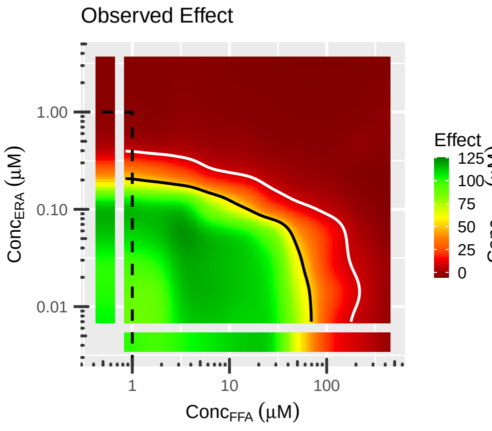

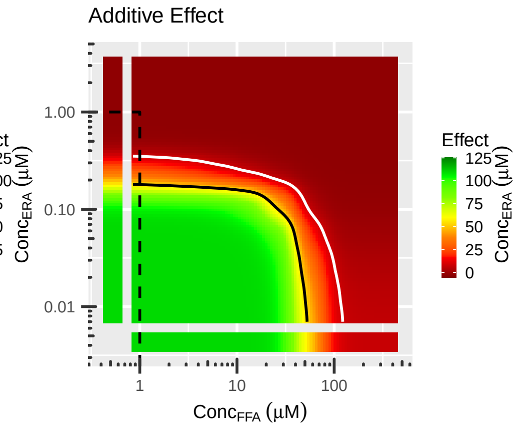

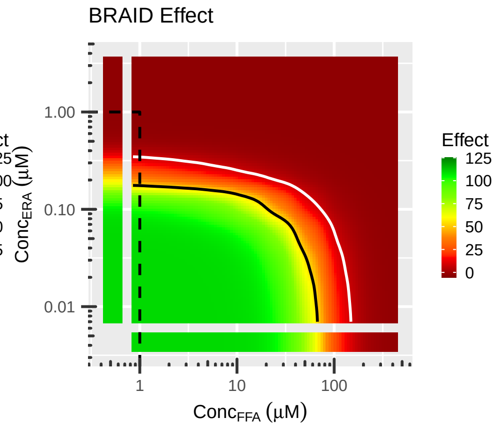

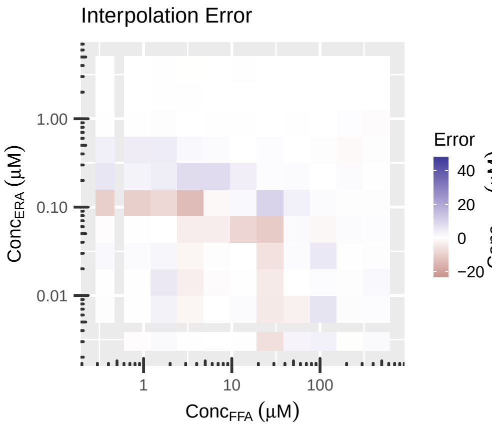

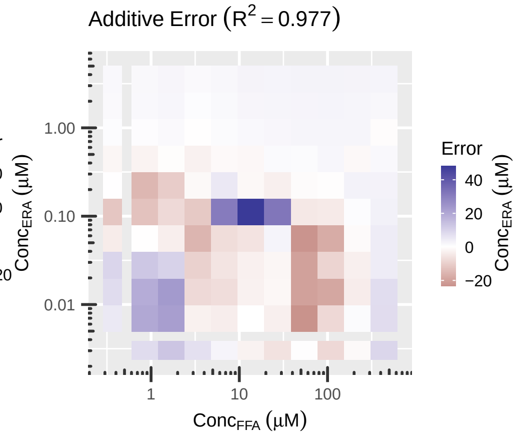

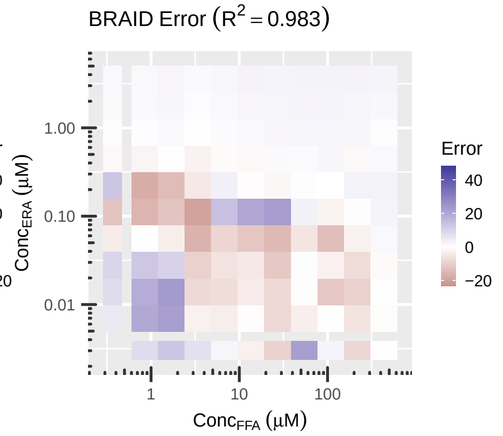

EC_{50} of FFA (μM)

ERA (μM)	Est
0	70.5 (64.9 – 76.1)
0.01	63.9 (60.4 – 67.4)
0.1	23.1 (21.2 – 25.3)
1	0 (0 – 0)

EC_{50} of ERAV (μM)

FFA (μM)	Est
0	0.218 (0.208 – 0.229)
1	0.191 (0.185 – 0.197)
10	0.141 (0.136 – 0.146)
100	0 (0 – 0)

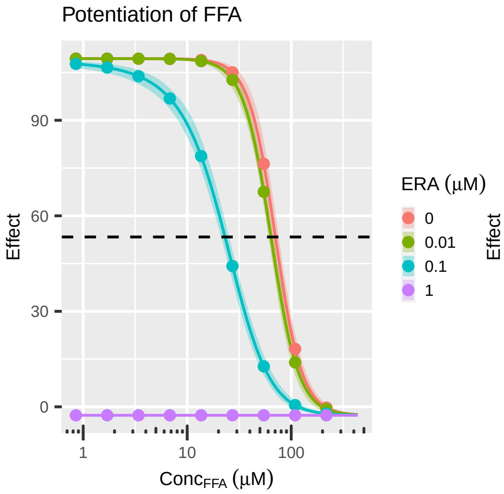

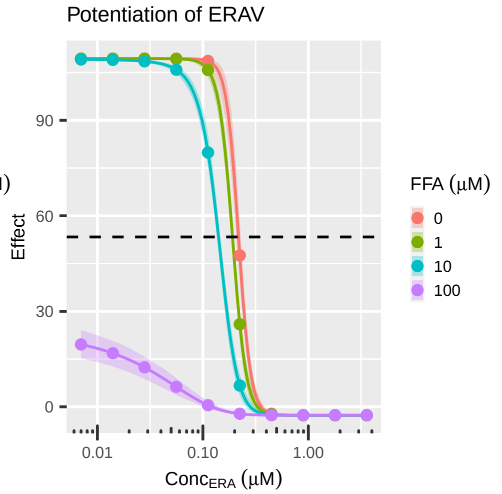

FFA vs. Linezolid

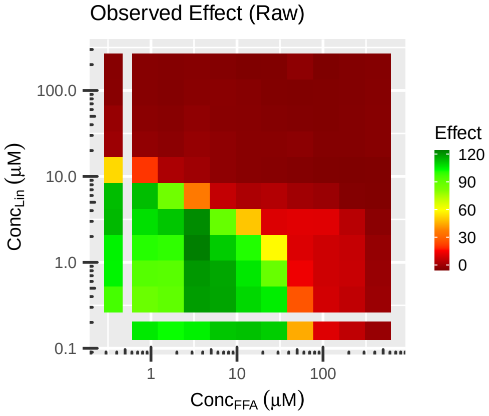

Best BRAID Fit

$ID_{M,A}$	54.6 (51.9 – 58.1)
$ID_{M,B}$	11.8 (11.5 – 12.1)
n_a	4.59 (4.16 – 5.04)
n_b	10 (8.76 – 10)
E_0	108.3274
$E_{f,A}$	5.28 (1.53 – 10.1)
$E_{f,B}$	–1.87752
κ	2.16 (2 – 2.3)

Additive Indices

IAE_{50}	1 (1 – 1)
IAE_{90}	1 (1 – 1)

BRAID Indices

IAE_{50}	1 (1 – 1)
IAE_{90}	1 (1 – 1)

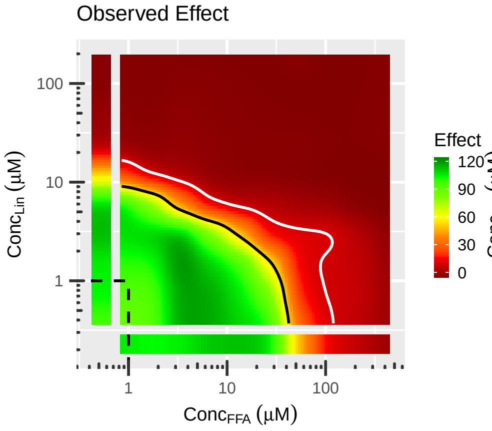

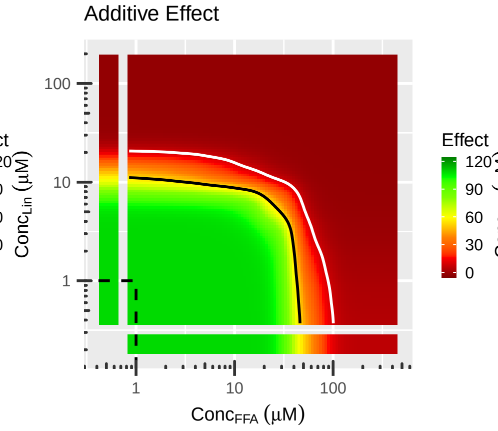

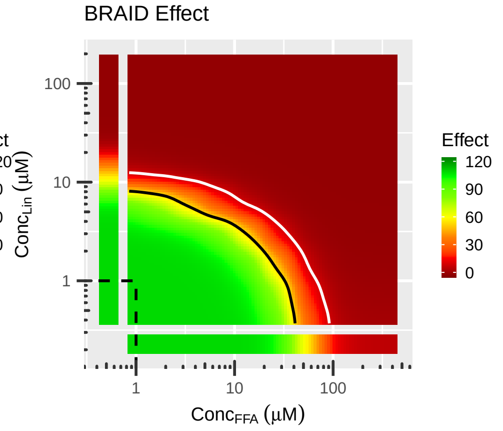

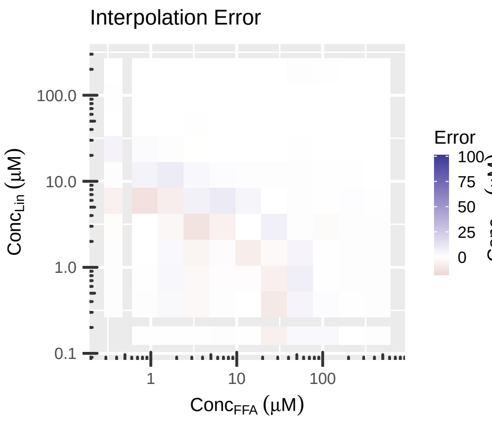

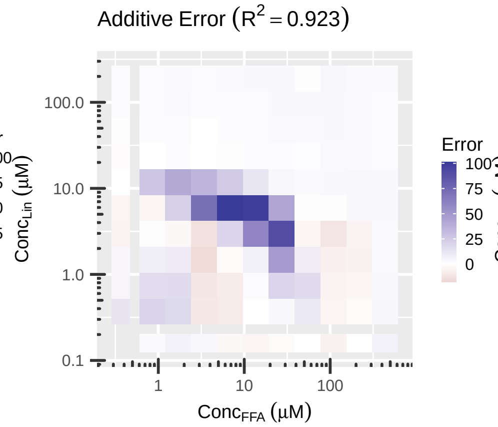

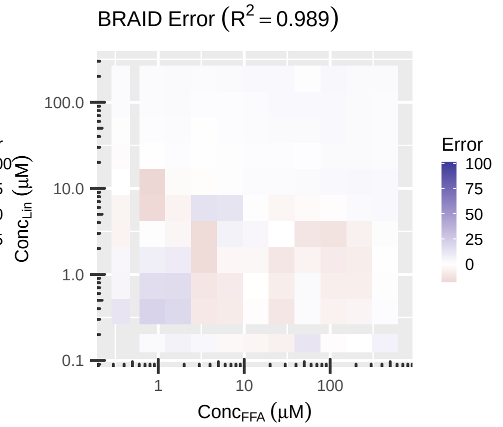

EC_{50} of FFA (μM)

Lin (μM)	Est
0	56.3 (53.6 – 59.8)
1	31.7 (30.7 – 32.9)
10	0.0786 (0.0554 – 0.122)
100	0 (0 – 0)

EC_{50} of Linezolid (μM)

FFA (μM)	Est
0	11.8 (11.5 – 12.1)
1	7.69 (7.55 – 7.96)
10	3.66 (3.58 – 3.8)
100	0 (0 – 0)

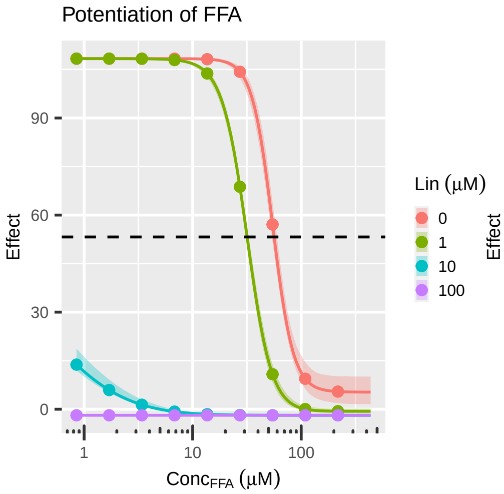

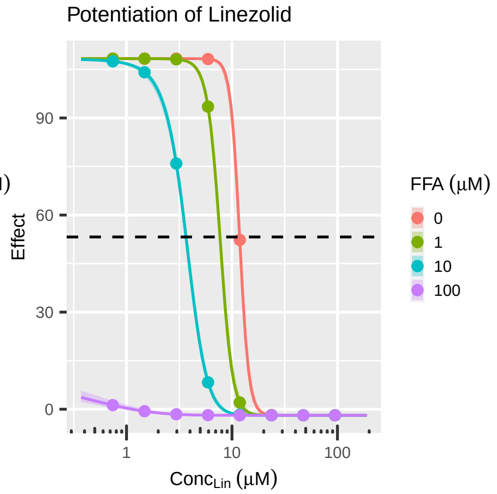

Supplement: Supplementary file 7 — BRAID models for each antibiotic in combination with FF-NH2. [file 41564_2025_2147_MOESM7_ESM.pdf]
